# Development, validation and application of single molecule molecular inversion probe based novel integrated genetic screening method for 29 common lysosomal storage disorders in India

**DOI:** 10.1186/s40246-024-00613-9

**Published:** 2024-05-10

**Authors:** Harsh Sheth, Aadhira Nair, Riddhi Bhavsar, Mahesh Kamate, Vykuntaraju K. Gowda, Ashish Bavdekar, Sandeep Kadam, Sheela Nampoothiri, Inusha Panigrahi, Anupriya Kaur, Siddharth Shah, Sanjeev Mehta, Sujatha Jagadeesan, Indrani Suresh, Seema Kapoor, Shruti Bajaj, Radha Rama Devi, Ashka Prajapati, Koumudi Godbole, Harsh Patel, Zulfiqar Luhar, Raju C. Shah, Anand Iyer, Sunita Bijarnia, Ratna Puri, Mamta Muranjan, Ami Shah, Suvarna Magar, Neerja Gupta, Naresh Tayade, Ajit Gandhi, Ajit Sowani, Shrutikaa Kale, Anil Jalan, Dhaval Solanki, Ashwin Dalal, Shrikant Mane, C. Ratna Prabha, Frenny Sheth, Chaitanya G. Joshi, Madhvi Joshi, Jayesh Sheth

**Affiliations:** 1 FRIGE Institute of Human Genetics, FRIGE House, Jodhpur Village Road, Satellite, Ahmedabad India 380015; 2 KLES Prabhakar Kore Hospital, Belgaum, Karnataka India; 3 Department of Pediatric Neurology, Indira Gandhi Institute of Child Health, Bangalore, India; 4 Department of Pediatrics, K.E.M Hospital, Pune, India; 5 Department of Paediatrics, Amrita School of Medicine, Kochi, India; 6 Postgraduate Institute of Medical Education and Research (PGIMER), Chandigarh, India; 7 Royal Institute of Child Neurosciences, Vastrapur, Ahmedabad India; 8 Department of Clinical Genetics and Genetic Counselling, Mediscan Systems, Chennai, India; 9 Division of Genetics and Metabolism Department of Pediatrics, Lok Nayak Hospital and Maulana Azad Medical College, New Delhi, India; 10 The Purple Gene Clinic, Simplex Khushaangan, SV Road, Malad West, Mumbai, India; 11 Rainbow Children’s Hospital, Hyderabad, India; 12 Genetic Care Clinic, Ahmedabad, India; 13 Deenanath Mangeshkar Hospital &Amp; Research Centre, Pune, India; 14 Zydus Hospital & Healthcare Research Pvt Ltd, Ahmedabad, India; 15 Civil Hospital, Asarwa, Ahmedabad India; 16 Ankur Institute of Child Health, Ahmedabad, India; 17 Neurokids Clinic, Ahmedabad, India; 18 Institute of Medical Genetics and Genomics, Sir Ganga Ram Hospital, New Delhi, India; 19 Department of Paediatrics, KEM Hospital, Parel, Mumbai India; 20 BJ Wadia Hospital for Children, Parel, Mumbai India; 21 MGM Medical College, Aurangabad, India; 22 Division of Genetics, Department of Pediatrics, All India Institute of Medical Sciences, New Delhi, India; 23 Department of Pediatrics, Dr. Panjabrao Deshmukh Memorial Medical College, Amravati, India; 24 Unique Hospital, Solapur, India; 25 NIRMAN, Vashi, India; 26 Mantra Child Neurology and Epilepsy Hospital, Bhavnagar, India; 27 Diagnostics Division, Centre for DNA Fingerprinting and Diagnostics, Hyderabad, India; 28 Department of Genetics, Yale School of Medicine, Yale Center for Genome Analysis, West Haven, CT USA; 29 Department of Biochemistry, Faculty of Science, The M. S. University of Baroda, Vadodara, India; 30 Gujarat Biotechnology Research Centre, Gandhinagar, Gujarat India

**Keywords:** Lysosomal storage disorders, smMIP probes, Dried blood spot, Diagnostic yield, Cost effective

## Abstract

**Background:**

Current clinical diagnosis pathway for lysosomal storage disorders (LSDs) involves sequential biochemical enzymatic tests followed by DNA sequencing, which is iterative, has low diagnostic yield and is costly due to overlapping clinical presentations. Here, we describe a novel low-cost and high-throughput sequencing assay using single-molecule molecular inversion probes (smMIPs) to screen for causative single nucleotide variants (SNVs) and copy number variants (CNVs) in genes associated with 29 common LSDs in India.

**Results:**

903 smMIPs were designed to target exon and exon–intron boundaries of targeted genes (n = 23; 53.7 kb of the human genome) and were equimolarly pooled to create a sequencing library. After extensive validation in a cohort of 50 patients, we screened 300 patients with either biochemical diagnosis (n = 187) or clinical suspicion (n = 113) of LSDs. A diagnostic yield of 83.4% was observed in patients with prior biochemical diagnosis of LSD. Furthermore, diagnostic yield of 73.9% (n = 54/73) was observed in patients with high clinical suspicion of LSD in contrast with 2.4% (n = 1/40) in patients with low clinical suspicion of LSD. In addition to detecting SNVs, the assay could detect single and multi-exon copy number variants with high confidence. Critically, Niemann-Pick disease type C and neuronal ceroid lipofuscinosis-6 diseases for which biochemical testing is unavailable, could be diagnosed using our assay. Lastly, we observed a non-inferior performance of the assay in DNA extracted from dried blood spots in comparison with whole blood.

**Conclusion:**

We developed a flexible and scalable assay to reliably detect genetic causes of 29 common LSDs in India. The assay consolidates the detection of multiple variant types in multiple sample types while having improved diagnostic yield at same or lower cost compared to current clinical paradigm.

**Supplementary Information:**

The online version contains supplementary material available at 10.1186/s40246-024-00613-9.

## Background

Lysosomal storage disorders (LSDs) are a group of ~ 70 monogenic metabolic disorders caused due to defect in the genes encoding lysosomal proteins and is estimated to have a combined incidence 1 in 1500 to 7000 live births [1]. Genes associated with LSDs include acid hydrolases, integral membrane proteins, activators and transporters [2]. LSDs are characterized by unwanted accumulation of metabolic substrate inside lysosomes, leading to cellular dysfunction and/or cell death. Clinical symptoms in children with LSDs develop progressively over time resulting in a wide spectrum of manifestations with variable severity. Most LSDs manifest in early childhood, however, late-onset juvenile and adult forms have also been reported [2–4]. In view of the recent development of therapeutic strategies for LSDs like enzyme replacement therapy (ERT), stem cell transplantation and emerging gene therapy for many LSDs, early diagnosis of LSDs is of particular relevance [5, 6].

The present diagnostic approach for LSDs includes a primary clinical evaluation followed by biochemical screening, confirmatory enzyme tests based on the detection of accumulated substrates and genetic study [2]. This 3-step diagnostic pathway is considered as “gold standard” for LSD diagnosis. However, enzyme testing is not available for some LSD types like the Niemann pick type C, activator protein deficiency and some forms of neuronal ceroid lipofuscinosis [2]. Furthermore, the enzyme testing involves analysis of a single enzymatic reaction at a given time, therefore, the approach is iterative in nature, especially in cases whereby multiple LSDs have overlapping phenotypic presentation and require sequential rounds of enzymatic testing to identify the disease. Overall, this route is time-consuming and expensive leading to poor diagnostic yields and long time to diagnosis [7].

India has a significant burden of LSDs, as indicated by several independent groups [8]. There is a high prevalence of Gaucher disease followed by the mucopolysaccharidosis group and approximately 20 other LSDs [8]. However, there are several challenges in achieving an accurate and timely diagnosis of LSDs in India. One of the reasons being paucity of quality assured diagnostic labs for biochemical enzyme based assays in the country. Furthermore, a study by Agarwal et al. 2015 demonstrated that the median time to reach a final diagnosis after the disease onset was 14 months [7]. This diagnostic delay has prognostic as well as therapeutic implications. Hence, there is a significant scope and need for improvement in the diagnosis of LSDs in India.

Significant advancements in next generation sequencing (NGS) technologies has led to simultaneous investigation of multiple genes with high accuracy and reduced costs compared to traditional biochemical assays. Indeed, several studies have explored use of multigene NGS panels for genetic diagnosis of LSDs with improved diagnostic yield compared to biochemical assays [9–11]. However, these panels have low diagnostic yields for diseases whereby the causative gene has a high sequence similarity with its pseudogene or consists of low complexity sequence region [12, 13]. Furthermore, multiplex ligation probe dependent amplification (MLPA) is required to detect copy number variations (CNV) in cases whereby single nucleotide variants (SNVs) have been ruled out by NGS panels, thereby adding complexity and cost to the diagnostic pathway.

Previously, single molecule molecular inversion probe (smMIP) coupled with unique molecular barcode (UMB) based target capture protocol followed by NGS has been used to detect both germline and somatic SNVs, CNVs and indels with high accuracy [14–16]. The key characteristics of this technique include consensus variant calling through the use of UMB, low cost per sample, minimum input DNA requirement, and high flexibility to include and exclude genes in the target capture step as required [16]. The potential for this highly flexible and affordable methodology in clinical practice is underlined by its low per sample cost coupled with easily manageable and scalable protocol [16, 17]. This approach therefore is likely to aid in improving genetic diagnostics of LSDs, especially in low-middle income countries (LMICs) like India.

Therefore, we developed and validated smMIP based NGS assay targeting coding regions of 23 genes that are associated with 29 common LSDs in India [18–20]. Furthermore, we validated this assay for its use on germline DNA sample extracted from dried blood spots, in order to increase its utility in clinical settings whereby dried blood spot sample type is only available. We hypothesized that this approach could reverse the current clinical “gold standard” diagnostic algorithm for LSDs whereby, the smMIP-NGS assay could be used as a first-line genetic test in patients clinically suspected with one of the 29 common LSDs in India followed by a biochemical and enzyme test to confirm the molecular findings. This alternative approach could help in reducing the time to reach a diagnosis and help initiate treatment. In this paper, we validated the assay on positive control samples that had been diagnosed with currently used methods. In addition, we studied the diagnostic value of this assay in a cohort of 300 clinically suspected or enzymatically diagnosed patients with LSDs.

## Methods

### Gene selection

Previous studies by Sheth et al. 2014 and Verma et al. 2012 have addressed the burden of LSDs in India and identified the most common LSDs [19, 20]. The selection of genes followed the American College of Medical Genetics (ACMG) guidelines on gene panel design for diagnostic purposes and reporting [21]. Based on the disease prevalence estimates, we included genes for seven classes of LSDs-sphingolipidoses, mucopolysaccharidosis, neuronal ceroid lipofuscinoses, integral membrane protein disorders, post-translational modification defects, activator protein deficiency and glycogen storage disorder. A total set of 23 genes with known association with the shortlisted LSDs were selected (Table 1).

**Table 1:** Overview of the 23 LSD genes included in the study and the percentage-coding region covered by the smMIP-assay

Gene name	Transcript	Disease name	Enzyme/protein	Diagnostic biomarker	Disease OMIM	Chromosome	Percentage coding region covered
*ARSA*	NM_000487.5	Metachromatic leukodystrophy	Arylsulphatase A	NA	250100	Chromosome 22	100
*ARSB*	NM_000046.3	MPS VI/Maroteaux–Lamy syndrome	Arylsulphatase B	Glycosaminoglycan (chondroitin sulfate, dermatan sulfate)	253200	Chromosome 5	100
*GAA*	NM_000152.3	Pompe disease	Glucosidase, alpha; acid	Tetrasaccharide glucose (Glc4)	232300	Chromosome 17	100
*GALC*	NM_001201401.1	Krabbe disease	Galactosylceramidase	Galactosylsphingosine/psychosine	245200	Chromosome 14	100
*GALNS*	NM_000512.4	MPS IV A/Morquio-A disease	Galactosamine (N-acetyl)-6-sulfate sulfatase	Glycosaminoglycan (keratin sulfate, chondroitin sulfate)	253000	Chromosome 16	98
*GBA*	NM_000157.3	Gaucher disease	Glucosidase, beta, acid	Chitotriosidase (ChT)	230800	Chromosome 1	91.5
*GLA*	NM_000169.2	Fabry disease	Galactosidase, alpha	Globotriaosylsphingosine (LysoGb3)	300644	Chromosome X	100
*GLB1*	NM_000404.2	GM1 gangliosidosis, MPS IV B	Galactosidase, beta 1	NA	230500	Chromosome 3	100
*GNPTAB*	NM_024312.4	Mucolipidosis II, III- alpha,beta	N-acetylglucosamine-1-phosphate transferase, alpha and beta subunits	NA	252500/255600	Chromosome 12	100
*HEXA*	NM_000520.4	Tay-Sachs disease	Hexosaminidase A (alpha polypeptide)	NA	272800	Chromosome 15	100
*HEXB*	NM_000521.3	Sandhoff disease	Hexosaminidase B (beta polypeptide)	NA	268800	Chromosome 5	100
*IDS*	NM_001166550.1	MPS II/Hunter syndrome	Iduronate 2-sulfatase	Glycosaminoglycan (chondroitin sulfate, dermatan sulfate, heparan sulfate)	309900	Chromosome X	98
*IDUA*	NM_000203.3	MPS I/Hurler syndrome	Iduronidase, alpha-L-	Glycosaminoglycan (chondroitin sulfate, dermatan sulfate, heparan sulfate)	252800	Chromosome 4	96
*NAGLU*	NM_000263.3	MPS III B/Sanfilippo B	N-acetylglucosaminidase, alpha	Glycosaminoglycan (chondroitin sulfate, heparan sulfate)	252920	Chromosome 17	100
*NPC1*	NM_000271.4	Niemann-pick disease type-C1	Niemann-Pick C1 protein	N-palmitoyl-O-phosphocholineserine (lyso-SM-509)	257220	Chromosome 18	100
*NPC2*	NM_006432.3	Niemann-pick disease type-C2	Niemann-Pick C2 protein	NA	601015	Chromosome 14	100
*PSAP*	NM_002778.2	Metachromatic leukodystrophy activator protein, Gaucher disease, atypical Krabbe disease, atypical Combined SAP deficiency	Prosaposin	Sulfatides in urine sample	619491, 611721, 610539, 611722, 249900	Chromosome 10	100
*SGSH*	NM_000199.3	MPS III A/Sanphilippo A	N-sulfoglucosamine sulfohydrolase	Glycosaminoglycan (chondroitin sulfate, heparan sulfate)	252900	Chromosome 17	100
*SMPD1*	NM_001007593.2	Niemann-pick disease type A&B	Sphingomyelin phosphodiesterase 1, acid lysosomal	Lysosphingomyelin (Lyso-SPM)	257200	Chromosome 11	97.5
*TPP1*	NM_000391.3	CLN-2 disease	Tripeptidyl peptidase I	NA	204500	Chromosome 11	100
*CLN6*	NM_017882.2	CLN-6 disease	Ceroid-lipofuscinosis, neuronal 6, late infantile, variant	NA	601780	Chromosome 15	100
*SLC17A5*	NM_012434.4	Sialic acid storage disease	Solute carrier family 17 (acidic sugar transporter), member 5	NA	604369/269920	Chromosome 6	100
*PPT1*	NM_000310.3	CLN-1 disease	Palmitoyl-protein thioesterase 1	NA	256730	Chromosome 1	100

### Patient cohort

*Validation cohort:* A total of 50 anonymized genomic DNA samples of patients with prior confirmed diagnosis with one of the 29 LSDs at FRIGE Institute of Human Genetics between 2008 and 2018 were obtained. All patients were diagnosed through biochemical and genetic tests. Genetic diagnosis was carried out using Sanger sequencing in 44 cases, MLPA in 4 cases and clinical exome sequencing in 2 cases.

*Diagnostic yield cohort:* We enrolled a total of 300 patients which were divided into two groups. The first group comprised of 187 patients which had only biochemical diagnosis for a given LSD. The second group comprised of 113 patients with a high clinical suspicion for one of the 29 common LSDs but no prior biochemical or genetic test was carried out. For all patients, genomic DNA was extracted from the peripheral blood sample of the patients by salting out protocol [22].

The ethics committee of the Foundation for Research in Genetics and Endocrinology (FRIGE) approved the study at the Institute of Human Genetics (Reg No- E/13237). The study comprised DNA samples of patients referred from Institute of Human Genetics, Ahmedabad as well as from other hospitals/reference laboratory/consultants across the country (Additional file 1). A written informed consent for the study was obtained from the guardians of all the participating subjects as per the Helsinki declaration.

### DNA extraction from dried blood spot (DBS)

Three patient samples whereby genetic diagnosis of LSD was carried out using the smMIP based assay in the germline DNA from EDTA treated peripheral blood sample were selected. Fresh peripheral blood sample was spotted on the dried blood spot (DBS) cards (HiMedia, India). DNA extraction from DBS cards was carried out in accordance with the InstaDNA kit protocol (HiMedia, India), with minor modifications. Briefly, 12 card punches of 3 mm size each was taken. The initial procedure was carried out in a petri plate for even distribution of wash with 1.5 ml of distilled water for 5 min. This was followed by transferring of the punch cards to 1.5 ml Eppendorf tubes containing 10 µl of proteinase K and 300 µl of wash solution. The sample was incubated at 65 °C for 15 min in a shaking incubator to facilitate cell lysis and DNA release. The supernatant was discarded, and the punch cards were washed with 300 µl of wash solution. A second wash was performed using 400 µl of TE buffer to rinse traces of salts and ethanol and the supernatant was subsequently discarded. The punch cards were then transferred to a petri plate for uniform drying at 65 °C for 15 min. Once dried, the punch cards wete transferred to a sterile tube containing 50 µl of Solution ID1. After pulse vortexing, the tubes were incubated at 65 °C for 15 min. Finally, 100 µl of ID2 solution was added and the sample was incubated at room temperature for 10 min. Extracted genomic DNA sample in the supernatant was collected in a sterile tube which was stored at − 20 °C. All genomic DNA samples were quantified using QIAexpert (Qiagen, Germany) and Qubit (Themo Fisher Scientific, USA).

### smMIP design

All smMIPs targeting the exons and intron–exon boundaries of the 23 genes were designed using the MIPGEN pipeline [23] and hg19/GRCh37 human reference genome build. The smMIP probe consisted of a 30 bp common linker arm containing a 5 bp random tag next to the extension arm. The random tag, also known as UMB, in the backbone of each MIP helps distinguish 1024 (4^5^) unique genomic DNA equivalents. This helps reduce potential PCR errors through removal of PCR duplicates and result in high-quality reads that helps detect SNV and CNV with high accuracy. Each smMIP probe covered a 110 bp genomic region with a maximum and minimum overlap of 40 and 20 bp, respectively, with the adjacent smMIP. Combined, 903 smMIPs targeted approximately 53.7 kb of the human genome (Additional file 2).

### smMIP pooling and phosphorylation

All 903 smMIP probes were pooled at a final concentration of 0.1 µM followed by phosphorylation. smMIPs were phosphorylated using 20U of T4 Polynucleotide Kinase (New England Biolabs, USA), 1X T4 DNA ligase buffer (New England Biolabs, USA), 50 µl of 0.1 µM of pooled smMIPs in a total reaction volume of 60 µl and incubated at 37֯ °C for 45 min followed by 65 °C for 20 min.

### smMIP capture, library preparation and sequencing

100 ng and 20 ng of genomic DNA from whole blood and DBS sample, respectively, was quantified using a Qubit dsDNA HS assay kit (Thermo Fisher Scientific, USA). It was subjected to smMIP capture in accordance with the protocol previously described with minor modifications [16, 24]. One hundred nanogram of genomic DNA was used as input and the target genomic regions were captured in a reaction containing smMIPs to genomic DNA in a molecular ratio of 1000:1. The capturing conditions were 95 °C for 10 min for denaturation of the double-stranded template DNA followed by 17 h of incubation at 60 °C. During this period, phosphorylated smMIPs hybridized against the single-stranded DNA followed by a gap-fill reaction and ligation to form circularized probes. All non-circularized probes and residual unused template DNA were digested in the following exonuclease treatment step. For amplifying the resultant circularized targets, 2X iProof Master Mix (BioRad, USA), common forward primer, and sample barcoded reverse primers were used. The thermal cycling conditions were as follows: 30 s at 98 °C followed by 19 cycles of 98 °C for 15 s, 60 °C for 30 s and 72 °C for 30 s, followed by 72 °C for 2 min. The primers used during this step contained adaptors compatible with Illumina sequencing platforms (Illumina, USA) [16]. The smMIP amplification products (269 bp) were analyzed on a 2% agarose gel.

After PCR, all the barcoded individual patient libraries were pooled together in equal volumes and purified using Agencourt AMPure XP beads as per the manufacturer’s protocol (Beckman Coulter, USA). The pooled and purified library was diluted to a concentration of 4 nM in 10 mM Tris EDTA (pH 8.5) and sequenced on Illumina MiSeq platform (Illumina, USA) using custom sequencing primers and 2 × 156 bp paired-end reads [16].

### Rebalancing the smMIP pool

In order to reduce the sequence coverage variability observed in the initial MIP experiment, the smMIP pool was rebalanced by adding a higher concentration (10x) of the underperforming smMIPs and an equimolar concentration of the unphosphorylated probes of overperforming smMIPs. The final concentration of each smMIP is provided in Additional file 2.

### Data analysis pipeline

All the FASTQ files containing the forward and reverse reads from all the samples were processed by trimming the 5 bp random tag from the reads and kept in key identifiers for later use. Following this, the reads were aligned to the hg19/GRCh37 human reference genome using BWA-MEM (v.0.7.12) [25] with the output presented as a sample specific BAM file amalgamated with the UMB data. Reads from the same smMIP i.e., containing the same UMB were discarded at random from the BAM file and the final coverage for individual smMIP was written to a coverage report. Single Nucleotide Variants (SNVs) were called using the GATK HaplotypeCaller (v4.1.2) following base quality score recalibration step, in accordance with the GATK best practice guidelines [26]. Variants were annotated, filtered and prioritized based on the patient’s phenotype (in HPO format) using Exomiser v12 [27] integrating data from SIFT (https://sift.bii.a-star.edu.sg/www/SIFT_seq_submit2.html), Polyphen2 (http://genetics.bwh.harward.edu/pph2), MutationTaster (http://www.mutationtaster.org), Combined Annotation Dependent Depletion (CADD) scores, REVEL scores, dbSNP (www.ncbi.nlm.nih.gov/SNP/), the Genome Aggregation Database (gnomAD; gnomad.broadinstitute.org) and ClinVar (www.ncbi.nlm.nih.gov/clinvar).

For detection of SNVs, BAM files of all the samples processed in a single sequencing run were used to normalize the coverage data and detect upto single-exon level CNVs. The CNV calls were carried out using DECoN v1.0.1 [28] using a minimum of 17 samples per batch for analysis.

### Variant validation and classification

Candidate variants identified in the patient samples were prioritized based on the minor allele frequency in the public databases, correlation of patient phenotype and biochemical report if available, predicted protein impact and predicted pathogenicity scores. All identified variants were assessed using Integrative Genomics Viewer (IGV) version 2.12.3 for read depth and read bias. Candidate SNVs were validated using specifically designed primers (https://bioinfo.ut.ee/primer3-0.4.0/) followed by Sanger sequencing for confirmation. The PCR products were purified using Exo-SAP-IT™ (USB Corporation, USA) and subjected to di-deoxy chain termination protocol using BigDye Terminator v3.1 cycle sequencing kit (Thermo Fisher Scientific, USA) and capillary electrophoresis was performed using an automated sequencer SeqStudio (Applied Biosystem, USA). Sequences were assessed by comparing with the hg19/GRCh37 genomic reference sequence of the specific genes using NCBI-BLAST (https://blast.ncbi.nlm.nih.gov/Blast.cgi). All candidate CNVs were validated using SYBR green dye (KAPA Biosystems, USA) based Q-PCR with ROX dye as a passive reference dye. Q-PCR reaction was carried out on the StepOne thermal cycler (Applied Biosystems, USA). Briefly, the reference gene used for the Q-PCR assay was *ALB*. Relative quantification approach was utilized whereby the Ct value was used as a determinant of the differences in the number of copies of the target sequence in different samples. Relative quantification (RQ) value of 0.5, 1 and 1.5 was suggestive of 1, 2 and 3 copies of the target sequence, respectively.

Finally, pathogenicity of the variants was classified according to the ACMG-AMP guidelines and ClinGen framework [29–31].

### Whole exome sequencing

Genomic DNA of 24 patients in whom no genetic diagnosis could be made using smMIP based assay was subjected to selective capture and sequencing of the protein coding regions using Human Core Exome enrichment kit (Twist Biosciences, USA). The prepared library was subjected to paired-end sequencing with a mean coverage of > 80-100X on the Illumina NovaSeq 6000 platform (Illumina, USA). FASTQ files were aligned against human reference genome GRCh37/hg19 using BWA MEM v0.7.12 [25]. SNVs and indels were called using GATK v4.12 HaplotypeCaller [26]. Additionally, CNVs were called using ExomeDepth v1.1.10 [32]. Variant annotation, filtration and prioritization was carried out using Exomiser v12.1.0 [27].

### Whole genome sequencing

Whole genome sequencing was performed for 7 cases at the Yale Centre for Genome Analysis whereby the cases were diagnosed with one of the 29 LSDs through biochemical assay but no genetic cause was identified in the smMIP based assay. 0.5ug of genomic DNA was enzymatically fragmented and end-repaired in a single reaction using xGen™ DNA EZ Library Prep Kit (IDT, USA). Size of the final library construct was determined on Caliper LabChip GXsystem and quantification was performed by Q-PCR SYBR Green reactions with a set of DNA standards using the Kapa Library Quantification Kit (KAPA Biosystems, USA). Libraries were sequenced on the Illumina NovaSeq 6000 platform and S4 flow cells with 2 × 150 bp paired-end reads and with yield of at least 700Gbp passing filter data per lane. Sample demultiplexing was carried out using Illumina’s bcl2dastq tool. Read alignment against hg38 human reference genome build and quality metrics were automatically generated using BWA-MEM/Picard pipeline and reviewed through the Yale Centre for Genome Analysis Tracking System software. Alignment and variant calling in the WGS data followed GATK v4 best practice guidelines. Variant annotation, filtration and prioritization was carried out using Exomiser v12.1.0 [27].

## Results

### Gene coverage descriptive statistics using smMIPs

A total of 903 smMIPs capturing the exons and intron–exon boundaries of 23 genes associated with 29 common LSDs were successfully designed (Additional file 2). We assessed the preliminary analytical performance of the assay in test samples (n = 3) that were previously genetically diagnosed for a given LSD. We analyzed the intra- and inter-sample uniformity of sequence coverage across all 23 genes. We sequenced 99.2% of the targeted region of approximately 53.7 kb with a mean (median) coverage after duplicate read removal of 536x (209x) during the first sequencing run. 147 smMIPs gave less than 30 reads during the first run. Upon rebalancing the probe pool, whereby tenfold increase in concentration of smMIPs having less than 30 reads in the first run was carried out, the mean (median) coverage was 442x (361x) with only 54 probes having less than 30 reads (Additional file 3). Approximately 0.8% of the targeted region which consisted of 5 genes- *GALNS* (2%), *GBA* (8.5%), *IDS* (2%), *IDUA* (4%), and *SMPD1* (2.5%), was not covered by any smMIP probes. This was because of either sequence similarity with a known pseudogene or presence of low-complexity sequence region. Specifically, exons 5 and 11 of the *GBA* gene have a high sequence similarity with the pseudogene *GBAP1*, which were analysed by Sanger sequencing in samples with clinical suspicion of Gaucher disease. Of the 350 DNA samples tested, all were successfully processed at the first sequencing effort leading to a high-quality sequencing result for 100% of the samples tested in the validation and diagnostic yield cohort.

### Assay validation

First, we evaluated the accuracy of our assay in detecting SNVs and CNVs by screening a cohort of 50 patients with known genetic aberrations, who were diagnosed using biochemical assay followed by conventional Sanger sequencing of a given LSD gene (Table 2). smMIP assay data analysis was done in a blinded fashion, and only after the result of the smMIP assay was interpreted, we compared the results to those of the conventional methods.

**Table 2:** Results obtained using smMIP assay in samples with previous genetic diagnosis for a particular LSD

Sr no	Patient ID	Gene	Disease	Transcript	Codon change (amino-acid change)	Zygosity	Concordant/discordant with previous diagnosis	Remarks
1	Sample 37	*IDUA*	MPS I/Hurler syndrome	NM_000203.5	c.757G > T (p.Gly253Cys)	HT	Concordant	
2	Sample 1	*IDS*	MPS II/Hunter syndrome	NM_000202.8	c.196C > T (p.Gln66*)	HEM	Concordant	
3	Sample 2	*IDS*	MPS II/Hunter syndrome	NM_000202.8	c.442G > A (p.Asp148Asn)	HEM	Concordant	
4	Sample 3	*IDS*	MPS II/Hunter syndrome	NM_000202.8	c.120_122del (p.Leu41del)	HEM	Concordant	
5	Sample 40	*IDS*	MPS II/Hunter syndrome	NM_000202.8	c.196C > T (p.Gln66Ter)	HEM	Discordant, Exon4-7 deletion detected	Deletion confirmed by end-point PCR
6	Sample 4	*GALNS*	MPS IV A/Morquio-A disease	NM_000512.5	c.230C > G (p.Pro77Arg)	HM	Concordant	
7	Sample 5	*GALNS*	MPS IV A/Morquio-A disease	NM_000512.5	c.107T > G (p.Leu36Arg)	HM	Variant missed	*smMIP doesn’t cover Exon 1 of the *GALNS* gene
8	Sample 6	*ARSB*	MPS VI/Maroteaux–Lamy syndrome	NM_000046.5	c.944G > T (p.Arg315Leu)	HM	Concordant	
9	Sample 7	*ARSB*	MPS VI/Maroteaux–Lamy syndrome	NM_000046.5	c.352_365dup (p.Pro123Serfs*16)	HM	Concordant	
10	Sample 41	*ARSB*	MPS VI/Maroteaux–Lamy syndrome	NM_000046.5	c.533A > T (p.His178Leu) c.944G > T (p.Arg315Leu)	HT	Concordant	
11	Sample 33	*GBA*	Gaucher disease	NM_000157.4	c.1177C > G (p.Leu393Val)	HM	Concordant	
12	Sample 34	*GBA*	Gaucher disease	NM_000157.4	c.721G > A (p.Gly241Arg)	HM	Concordant	
13	Sample 48	*GBA*	Gaucher disease	NM_000157.4	c.1060G > A (p.Asp354Asn)	HM	Concordant	
14	Sample 10	*SMPD1*	Niemann-pick disease type A&B	NM_000543.5	c.1699C > T (p.Gln567*)	HM	Concordant	
15	Sample 11	*SMPD1*	Niemann-pick disease type A&B	NM_000543.5	c.1624C > T (p.Arg542*)	HM	Concordant	
16	Sample 14	*ARSA*	Metachromatic leukodystrophy	NM_000487.6	c.731G > A (p.Arg244His)	HM	Concordant	
17	Sample 15	*ARSA*	Metachromatic leukodystrophy	NM_000487.6	c.979 + 1G > A	HM	Concordant	
18	Sample 16	*ARSA*	Metachromatic leukodystrophy	NM_000487.6	c.1130_1132del (p.Phe377del)	HM	Concordant	
19	Sample 39	*GALC*	Krabbe disease	NM_000153.4	30 Kb deletion c.908 + 1G > A	HT	Concordant	
20	Sample 22	*HEXA*	Tay-Sachs disease	NM_000520.6	c.1385A > T (p.Glu462Val)	HM	Concordant	
21	Sample 23	*HEXA*	Tay-Sachs disease	NM_000520.6	c.1385A > T (p.Glu462Val) exon 1 deletion	HT	Concordant	
22	Sample 24	*HEXA*	Tay-Sachs disease	NM_000520.6	c.1385A > T (p.Glu462Val) exon 1 deletion	HT	Concordant	
23	Sample 25	*HEXA*	Tay-Sachs disease	NM_000520.6	exon 2 and 3 deletion	HM	Concordant	
24	Sample 26	*HEXA*	Tay-Sachs disease	NM_000520.6	exon 2 and 3 deletion	HM	Concordant	
25	Sample 44	*HEXA*	Tay-Sachs disease	NM_000520.6	c.1385A > T (p.Glu462Val)	HM	Concordant	
26	Sample 27	*HEXB*	Sandhoff disease	NM_000521.4	c.611G > A (p.Gly204Glu)	HM	Concordant	
27	Sample 28	*HEXB*	Sandhoff disease	NM_000521.4	c.1550_1553dup (p.Asp518Glufs*8)	HM	Concordant	
28	Sample 45	*HEXB*	Sandhoff disease	NM_000521.4	c.1563_1573del (p.Met522LeufsTer2)	HM	Concordant	
29	Sample 12	*GLB1*	GM1 gangliosidosis	NM_000404.4	c.1077del (p.Val360Tyrfs*23)	HM	Concordant	
30	Sample 13	*GLB1*	GM1 gangliosidosis	NM_000404.4	c.65_75 + 1del (p.Arg22_Asn26delinsGln)	HM	Concordant	
31	Sample 43	*GLB1*	GM1 gangliosidosis	NM_000404.4	c.562G > T (p.Glu188Ter) c.1010T > C (p.Leu337Pro)	HT	Concordant	
32	Sample 8	*GAA*	Pompe disease	NM_000152.5	c.1A > G	HM	Concordant	
33	Sample 9	*GAA*	Pompe disease	NM_000152.5	c.1A > G c.1942G > A (p.Gly648Ser)	HT	Concordant	
34	Sample 31	*PPT1*	CLN-1 disease	NM_000310.4	c.133T > C (p.Cys45Arg)	HM	Concordant	
35	Sample 32	*PPT1*	CLN-1 disease	NM_000310.4	c.713C > T (p.Pro238Leu)	HM	Concordant	
36	Sample 46	*PPT1*	CLN-1 disease	NM_000310.4	c.541G > A (p.Val181Met)	HM	Concordant	
37	Sample 29	*TPP1*	CLN-2 disease	NM_000391.4	c.616C > T (p.Arg206Cys)	HM	Concordant	
38	Sample 30	*TPP1*	CLN-2 disease	NM_000391.4	c.1015C > T (p.Arg339Trp)	HM	Concordant	
39	Sample 47	*TPP1*	CLN-2 disease	NM_000391.4	c.616C > T (p.Arg206Cys)	HM	Concordant	
40	Sample 18	*GNPTAB*	Mucolipidosis II, III- alpha,beta	NM_024312.5	c.3335 + 1G > A	HM	Concordant	
41	Sample 19	*GNPTAB*	Mucolipidosis II, III- alpha,beta	NM_024312.5	c.3336-1G > A	HM	Concordant	
42	Sample 20	*GNPTAB*	Mucolipidosis II, III- alpha,beta	NM_024312.5	c.2693dup (p.Tyr899Valfs*21) c.3503_3504del (p.Leu1168Glnfs*5)	HT	Concordant	
43	Sample 49	*GNPTAB*	Mucolipidosis II, III- alpha,beta	NM_024312.5	c.2957G > A (p.Arg986His)	HM	Concordant	
44	Sample 50	*GNPTAB*	Mucolipidosis II, III- alpha,beta	NM_024312.5	c.3307_3318delAAAGCATATAAG insCAGTAACT (p.Lys1103Leufs*19)	HM	Concordant	
45	Sample 36	*NPC1*	Niemann-pick disease type-C1	NM_000271.5	c.3182 T > C (p.Ile1061Thr)	HT	Concordant and splice variant c.955 + 3A > G detected	A splice variant was detected which was previously not reported. Both variants were confirmed by Sanger sequencing
46	Sample 42	*NPC2*	Niemann-pick disease type-C2	NM_006432.5	c.141C > A (p.Cys47Ter)	HM	Concordant	
47	Sample 21	*NPC2*	Niemann-pick disease type-C2	NM_006432.5	c.82 + 2 T > C	HM	Concordant	
48	Sample 17	*CLN6*	Ceroid lipofuscinosis, neuronal, 6A	NM_017882.2	c.679G > A:p.(Glu227Lys)	HM	Concordant	
49	Sample 35	*PSAP*	Metachromatic leukodystrophy due to SAP-b deficiency	NM_002778.2	c.679_681del:p.(Lys227del)	HM	Concordant	
50	Sample 38	*SLC17A5*	Sialic acid storage disorder, infantile	NM_012434.4	c.116G > A:p.(Arg39His)	HM	Concordant	

HEM: Hemizygous, HM: homozygous, HT: heterozygous

^*^Exon 1 of GALNS: Sanger sequencing

Overall, our smMIP based assay gave concordant results with conventional methods in 98% of the cases (n = 49/50; Table 2). All of the SNVs in the previously diagnosed samples, except for one sample, were accurately identified by our assay. The single discordant case was “sample 5” where the variant c.107 T > G was not detected in the *GALNS* gene. The smMIP assay failed to detect this variant as the variant was present in a low-complexity region of the gene for which smMIP probes were not designed by the MIPgen tool. Of interest, the smMIP assay detected a multi-exon deletion in the *IDS* gene in “sample 40” which was previously not detected by conventional methods. The smMIP assay result was validated by end-point PCR. Additionally, in “sample 36”, the smMIP assay detected compound heterozygous variants c.955+3G>A and c.3182T>C in the *NPC1* gene. Interestingly, the prior variant was undetected by the conventional method, hence, the smMIP assay could provide complete genetic diagnosis for this sample.

We also assessed the strength of our assay in detecting CNVs. 10% of the samples (n = 5/50) had prior diagnosis of deletions in the *HEXA* gene (n = 4/5) and *GALC* gene (n = 1/5). Our smMIP-based assay correctly identified two homozygous *HEXA* deletions, two heterozygous *HEXA* deletions, and one heterozygous *GALC* deletion, which was consistent with the previous diagnosis (Table 2). Overall, our assay detected 97.9% SNVs and 100% CNVs in the validation cohort samples.

### Diagnostic yield in enzymatically confirmed cases of LSD

We assembled a cohort of 187 patients that had received diagnosis for one of the 29 common LSDs through biochemical tests only. The classification of all samples based on the LSD sub-types were as following: mucopolysaccharidoses (n = 60/187; 32.2%), sphingolipidoses (n = 89/187; 47.8%), glycogen storage disease (n = 7/187; 4%), neuronal ceroid lipofuscinoses (n = 14/187; 8%), and post-translational modification defects (n = 17/187; 8.6%). For all these patients, clinical data and biochemical enzyme test results were collected and are presented in Additional file 4 and was used to carry out data analysis of the smMIP-assay.

**Fig. 1:**
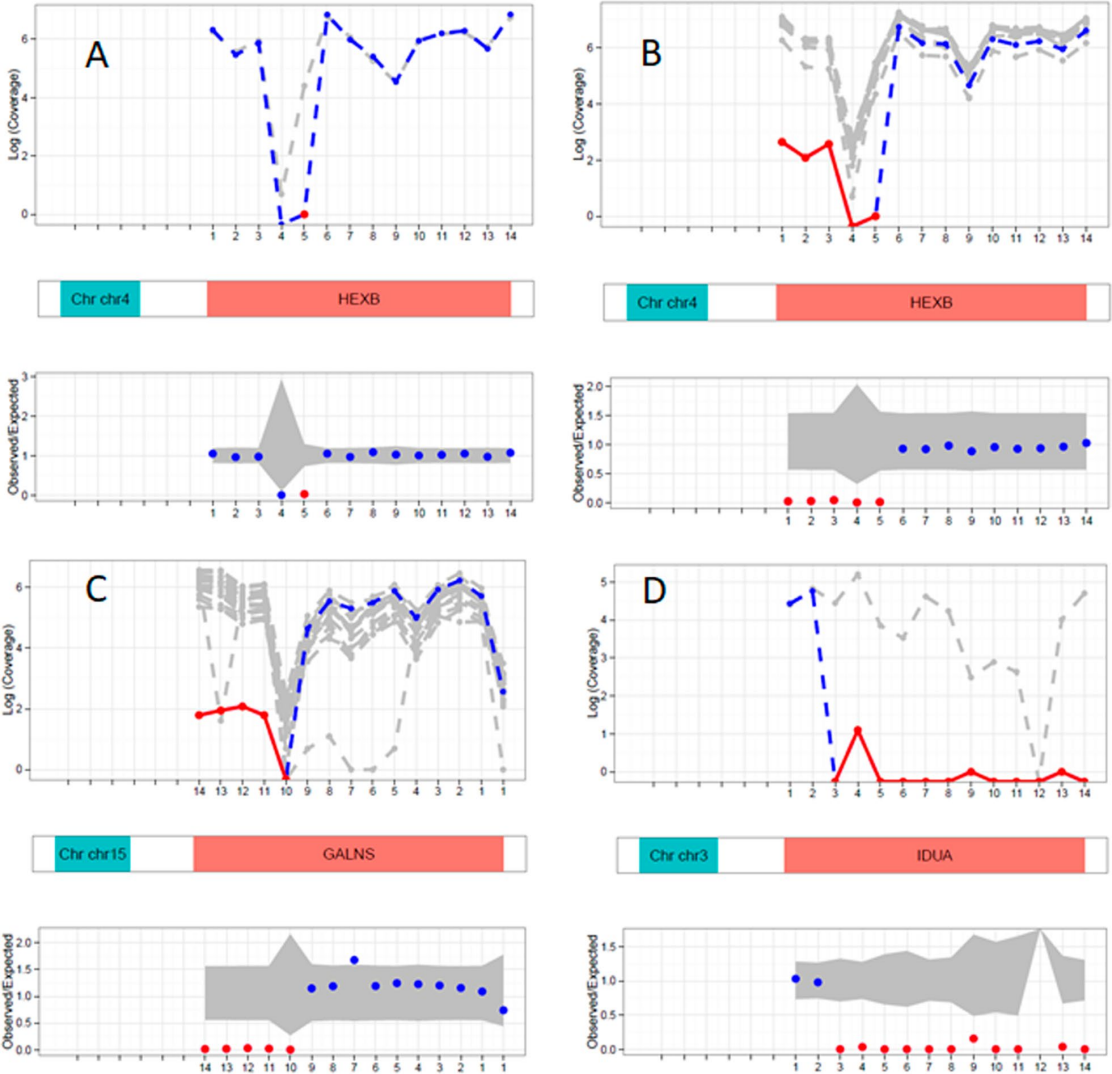
Automated visualizations of copy number variants from the DECoN tool. The top plot shows log normalised coverage of sample of interest (blue) relative to the reference samples (grey). Bottom plot shows ratio of observed to expected coverage. Relevant genes shown in between the plots in red. **A** Sample showing a homozygous exon 5 deleted in the *HEXB* gene. **B** Sample showing a homozygous exon 1–5 deleted in the *HEXB* gene. **C** Sample showing a homozygous exon 10–14 deleted in the *GALNS* gene. **D** Sample showing a homozygous exon 3–14 deleted in the *IDUA* gene. Deleted exons are highlighted in red

**Fig. 2:**
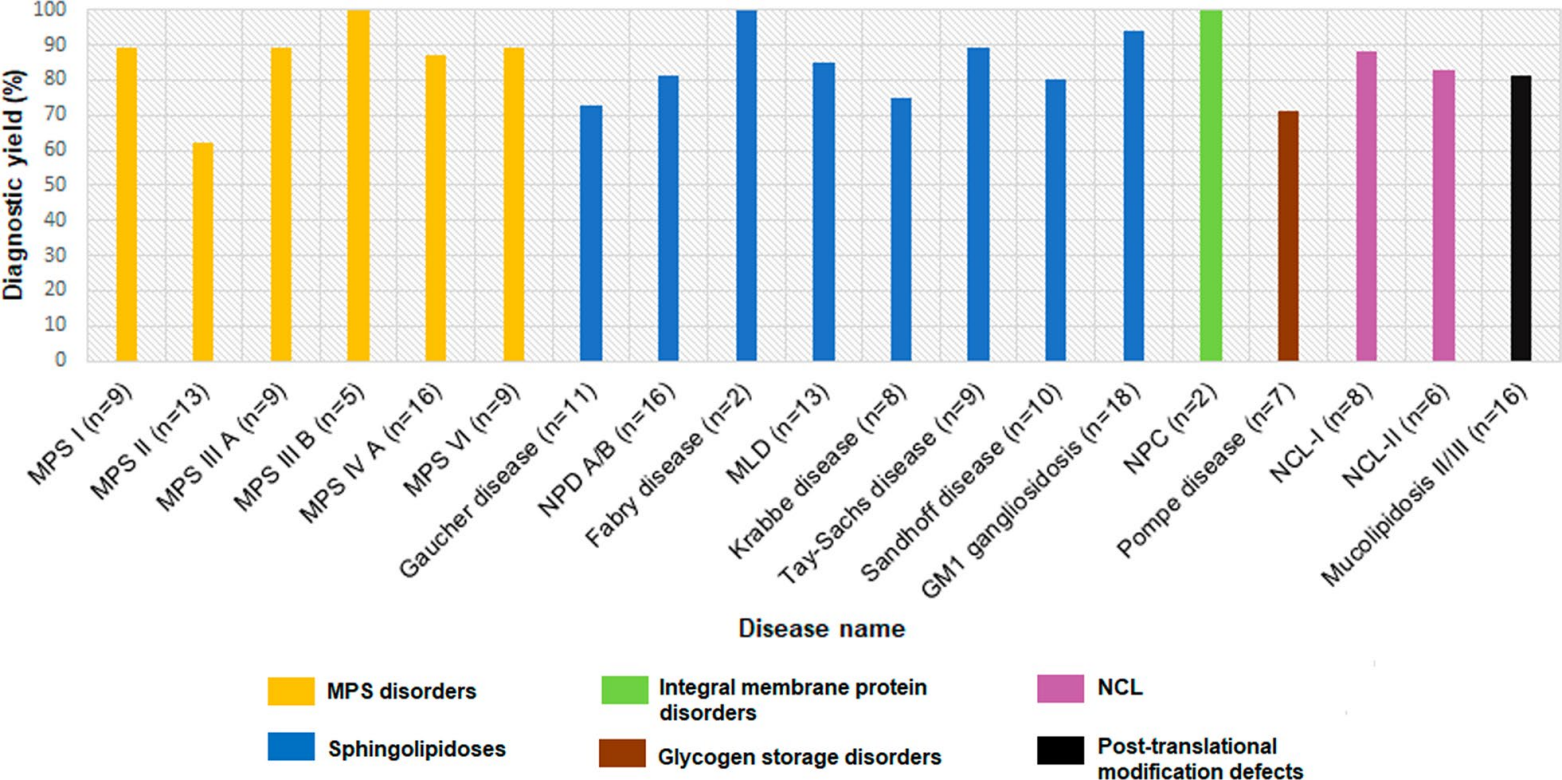
Diagnostic yield observed by smMIP-based NGS assay in the cohort with an enzyme diagnosis for a particular LSD

Our analysis led to a confirmed genetic diagnosis in 156 of the 187 enzymatically confirmed cases (83.4%) with the presence of pathogenic or likely pathogenic variants in the targeted regions. For SNVs, the smMIP assay could detect all types of variants in the targeted region, with missense variants being the predominant variant type- missense (63%), nonsense (9%), splice site (11%), and small insertions/deletions (17%). Of 156 cases with a confirmed genetic diagnosis, CNVs spanning from single to multiple exons were observed in 8 cases (5%). This included 4 multi-exon deletions: 2 in the *GALNS* gene, one in the *IDUA* gene, and an exon 1–5 deletion in the *HEXB* gene. Single exon deletions were detected in the *GALC*, *HEXB, IDS* and *PPT1* genes, one case of each (Fig. 1). Overall, we found CNVs in 5% of the total molecularly diagnosed cases in the present cohort. Of the 25 patients without molecular genetic diagnosis, we detected heterozygous pathogenic variant in the targeted regions of *GLB1* and *GNPTAB* genes in 2 patients. These patients were enzymatically diagnosed as GM1 gangliosidosis and Mucolipidosis II/III, respectively. However, a second heterozygous variant in *trans* was not detected by the smMIP-assay, possibly suggestive of the variant present in the deep intronic region or presence of a complex structural variant; both of which are not detectable by the present assay. Figure 2 shows overview of the diagnostic yield achieved by the smMIP-based assay across 19 out of the 29 LSDs studied. We observed a 100% diagnostic yield in cases with the following LSDs: MPS IIIB (n = 5/5), Fabry disease (n = 2/2), and Niemann-pick type-C (n = 2/2) and 94% diagnostic yield in GM1 gangliosidosis cases (n = 17/18).

The smMIP-based assay identified causative variant(s) in 8 out of 9 cases each with either MPS I, MPS IIIA, MPS VI and Tay-Sachs disease thereby giving a diagnostic yield of 89% in these disease types. Additionally, the assay had a diagnostic yield of 86.6%, 73%, 71.4% and 88% for MPS IVA, Gaucher disease, Pompe disease and Mucolipidosis II/III disease, respectively. In 15 cases where no causative variant(s) were identified by the smMIP-based assay, whole exome/genome sequencing was carried out in order to assess if the variant(s) were present in deep intronic regions or were complex structural variants which would be missed by the smMIP based assay. On analysis of 15 cases, only a single case could be resolved whereby the case was enzymatically diagnosed to have Tay-Sachs disease and a single heterozygous variant c.902T>G in the *HEXA* gene was previously detected with the smMIP based assay (Table 3). We detected a deep intronic heterozygous variant c.413-358del in intron 3 of the *HEXA* gene, which was in *trans* with the aforementioned variant in this case. However, due to the lack of in vivo functional evidence for the intronic variant, the variant was classified as a variant of uncertain significance.

**Table 3:** Whole genome/whole exome study in enzymatically diagnosed cases of LSD with negative result by the smMIP-based NGS assay

S. no.	Patient ID	Enzyme name (normal range)	Enzyme value (nmol/h/mg protein)	smMIP study	Whole genome study	Whole exome study	Additional variant identified
1	LSD11	α-iduronidase-sulfatase (400–1616 nmol/h/ml plasma)	6.3	No variant identified	Negative	–	–
2	LSD25	α-iduronidase-sulfatase (400–1616 nmol/h/ml plasma)	3.6	No variant identified	–	Negative for *IDS* gene	–
3	LSD33	α-iduronidase-sulfatase (400–1616 nmol/h/ml plasma)	0	No variant identified	–	Negative for *IDS* gene	–
4	LSD300	α-iduronidase-sulfatase (400–1616 nmol/h/ml plasma)	0	No variant identified	-	Negative for *IDS* gene	-
5	LSD182	Heparan sulfamidase (2.1–9.5 nmol/h/mg protein)	0	No variant identified	–	Negative for *SGSH* gene	Hemizygous for c.415G > A (p.Ala139Thr) in *TFE3* gene (Variant of uncertain significance)
6	LSD106	β-galactosidase-6-sulfate-sulfatase (2.8–42.6 nmol/h/mg protein)	0.23	No variant identified	Negative	–	–
7	LSD7	β-glucosidase (4.0–32.8 nmol/h/mg protein)	0.2	No variant identified	Negative	–	–
8	LSD45	β-glucosidase (4.0–32.8 nmol/h/mg protein)	0.52	No variant identified	Negative	–	–
9	LSD196	Sphingomyelinase (1.8–9.6 nmol/h/mg protein)	0.04	No variant identified	–	Negative for *SMPD1* gene	–
10	LSD64	Sphingomyelinase (1.8–9.6 nmol/h/mg protein)	0.55	No variant identified	–	Negative for *SMPD1* gene	–
11	LSD48	β-hexosaminidase-A (62.7–659.4 nmol/h/mg protein)		No variant identified	Heterozygous for c. 413-358del in intron 3 of the *HEXA* gene	–	–
12	LSD199	β-hexosaminidase-A (62.7–659.4 nmol/h/mg protein)	14.3	No variant identified	–	Negative for *HEXA* gene	–
13	LSD204	α-1,4-glucosidase (with acarbose/without acarbose: 0.29–0.68)	0.21	No variant identified	–	Negative for *GAA* gene	Homozygous for c.1850 T > C (p.Leu617Pro)in *ACOX1* gene (Variant of uncertain significance)
14	LSD116	I-cell screening	Positive	No variant identified	Negative	–	–
15	LSD76	I-cell screening	Positive	No variant identified		Negative	–

Poor diagnostic yield with the smMIP based assay was observed for MPS II cases whereby the yield was only 30.8% (n = 4/13). As the exon 3 of the *IDS* gene is not targeted by the smMIP-based assay due to its high sequence similarity with the pseudogene *IDSP1*, the remaining 8 cases were subjected to Sanger sequencing for exon 3. This led to diagnosis in 4 cases, leading to an overall genetic diagnosis in 62% of the MPS II cases (n = 8/13; Additional file 4). For the remaining 4 cases, no causative variants could be identified with whole exome sequencing (Table 3).

Of note, we observed 3 cases (sample ID: LSD1, LSD185 and LSD91) where there was discordance between the genetic diagnosis from smMIP based assay and biochemical assay. In 2 cases where the biochemical diagnosis of Gaucher disease was made due to the low levels of beta-glucosidase enzyme in leukocytes, the smMIP based assay detected pathogenic variant in the *NPC1* and *NPC2* gene in either case. In the third case with diagnosis of MPS I based on the biochemical assay, the smMIP-based assay detected no causative variants in the *IDUA* gene. In fact, a homozygous variant c.3503_3504del (p.Leu1168Glnfs*5) in the *GNPTAB* gene was observed which led to the genetic diagnosis of Mucolipidosis II/III. Thus, using the smMIP-based assay in all three cases, we could rectify the previous misdiagnosis of Gaucher disease as a case of Niemann Pick type C and MPS I as a case of Mucolipidosis II/III.

### Diagnostic yield in cases with a clinical suspicion of LSD

**Fig. 3:**
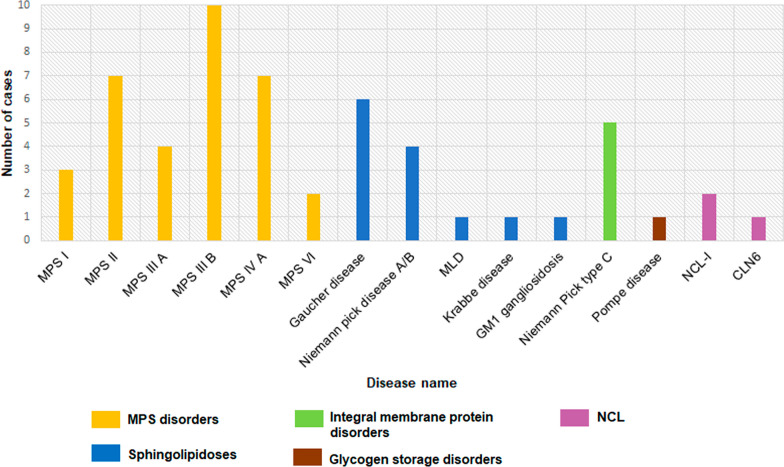
Disease wise distribution of patients diagnosed by smMIP-based NGS assay in the clinical suspicion cohort

We assessed 113 cases clinically suspected with one of the 29 LSDs using the smMIP-based assay (Additional file 5). We stratified these patients into “high-index” and “low-index” clinical suspicion groups based on the likelihood rank for one of the 29 LSDs using the phenotype scoring tool- GDDP (https://gddp.research.cchmc.org/). Patients where the rank was 1 to 15 were stratified to the “high-index” group (n = 73) and those with the rank > 15 were stratified to the “low-index” group (n = 40). A significantly higher diagnostic yield was observed in the high-index group (n = 54/73; 73.9%) compared to the low-index group (n = 1/41; 2.4%) using the smMIP-based assay (Additional file 5). The majority of the cases diagnosed in this entire cohort of patients belonged to mucopolysaccharidosis (n = 30); chiefly- MPS IIIB (n = 10), MPS II (n = 7), MPS IVA (n = 7), 4 cases of MPS IIIA, 3 cases of MPS I, and 2 cases of MPS VI. Using this assay, we identified causative variants in the *GBA* gene and *SMPD1* gene in six patients and four patients respectively (Fig. 3).

Importantly, with this assay we could provide a genetic diagnosis for Niemann pick type C and neuronal ceroid lipofuscinosis-6 for which currently, no biochemical tests are available. Overall, we identified 5 patients with Niemann pick type C, despite these patients being clinically suspected with Gaucher disease or Niemann-pick disease A/B.

Lastly, 17 cases in the high-index group where no causative variant(s) were identified using the smMIP based assay were subjected to WES (Table 4). Of note, 3 out of 17 cases were diagnosed with rare LSDs-Sialidosis type I/type II (OMIM#256550), Wolman disease (OMIM#620151) and GM2 gangliosidosis AB variant (OMIM#272750)-which are not covered by the smMIP based assay due to their low prevalence in the Indian population [19]. In further 7 cases, diseases not associated with LSDs were identified- progressive pseudorheumatoid dysplasia (OMIM#208230), intellectual developmental disorder 23 (OMIM#615761), hypermanganesemia with dystonia-1 (OMIM#613280), Neurodevelopmental disorder with or without hypotonia, seizures, and cerebellar atrophy (OMIM#616917), Beck-Fahrner syndrome (OMIM#618798) and microcephaly, short stature, and limb abnormalities (OMIM#617604) (Table 4).

**Table 4:** Whole exome sequencing in high-index clinical suspicion cases of LSDs undiagnosed by smMIP-based NGS assay

S. no.	Patient ID	Clinical suspicion by GDDP	smMIP study	Whole exome sequencing						Classification
				Gene	Transcript	Variant	Exon	Zygosity	Disease	
1	LSD158	NPD/Gaucher	No variant identified	NA	NA	NA	NA	NA	NA	NA
2	LSD159	GM1/GM2	No variant identified	*AGL*	ENST00000361915.8	c.1525A > G (p.Met509Val)	Exon 12	Heterozygous	Glycogen storage disease IIIa & IIIb (OMIM#232400)	Uncertain significance
3	LSD160	MPS IV	No variant identified	*CCN6*	ENST00000368666.7	c.296_298delinsTTA (p.Tyr99_Cys100delinsPheSer)	Exon 2	Homozygous	Progressive pseudorheumatoid dysplasia (OMIM#208230)	Likely Pathogenic
4	LSD174	GM1/GM2	No variant identified	*SETD5*	ENST00000402198.7	c.2734C > T (p.Arg912Ter)	Exon 19	Heterozygous	Intellectual developmental disorder 23 (OMIM#615761)	Pathogenic
5	LSD298	MPS IV	No variant identified	*NEU1*	ENST00000375631.5	c.679G > A (p.Gly227Arg)	Exon 4	Homozygous	Sialidosis, type I (OMIM#256550) /Sialidosis, type II (OMIM#256550)	Pathogenic
6	LSD299	Sialidosis	No variant identified	NA	NA	NA	NA	NA	NA	NA
7	LSD252	NPD/Gaucher	No variant identified	*SLC30A10*	ENST00000366926.4	c.1059T > A (p.Tyr353Ter)	Exon 4	Homozygous	Hypermanganesemia with dystonia-1 (OMIM#613280)	Pathogenic
8	LSD254	MPS	No variant identified	*PIGG*	ENST00000453061.7	c.2624_2625del (p.Leu875Ter)	Exon 12	Homozygous	Neurodevelopmental disorder with or without hypotonia, seizures, and cerebellar atrophy (OMIM#616917)	Pathogenic
9	LSD257	GM1/GM2	No variant identified	*TET3*	ENST00000409262.8	c.1503_1508del (p.Ala502_Pro503del)	Exon 4	Homozygous	Beck-Fahrner syndrome (OMIM#618798)	Uncertain significance
10	LSD258	GM1/GM2	No variant identified	*TET3*	ENST00000409262.8	c.1503_1508del (p.Ala502_Pro503del)	Exon 4	Homozygous	Beck-Fahrner syndrome (OMIM#618798)	Uncertain significance
11	LSD269	NPD/GM2	No variant identified	NA	NA	NA	NA	NA	NA	NA
12	LSD279	Oligosaccharidosis	No variant identified	NA	NA	chr1:g.(?_923456)_(3829997_?)del	NA	Heterozygous	–	Likely pathogenic
13	LSD283	NPD/Gaucher	No variant identified	*LIPA*	ENST00000336233.10	c.1033G > A (p.Asp345Asn)	Exon 10	Homozygous	Wolman disease (OMIM#620151)	Likely Pathogenic
14	LSD244	MPS	No variant identified	*DONSON*	ENST00000303071.10	c.-5_33del (3’ Start Loss)	Exon 1	Homozygous	Microcephaly, short stature, and limb abnormalities (OMIM#617604)	Uncertain significance
15	LSD270	NPD/GM2/GM1	No variant identified	*GM2A*	ENST00000357164.3	c.81 + 1delG	Intron 1	Homozygous	GM2-gangliosidosis, AB variant (OMIM#272750)	Likely Pathogenic
16	LSD265	GM1/GM2	No variant identified	NA	NA	NA	NA	NA	NA	NA
17	LSD277	NPD	No variant identified	NA	NA	NA	NA	NA	NA	NA

*NA* no variant identified

### Performance of smMIP based assay in DBS samples

Three patients whose blood sample was available for DBS and previously received genetic diagnosis using smMIP based assay was analysed for a comparative sequencing quality performance. Compared to 100 ng of input DNA extracted from blood, 20 ng of DNA was used for targeted capture and subsequent sequencing using smMIP based assay. Whilst no difference was observed in the percentage of mapped reads inside target region (96.9% for DNA from blood versus 97.4% for DNA from DBS), a higher proportion of duplicate reads based on UMB were detected (38% for DNA from blood versus 45% for DNA from DBS) (Additional file 6). With 80% reduction in the input DNA quantity, we observed a 38% drop in average sequence coverage across samples sequenced from DBS (160x) versus whole blood (258x) (Additional file 7). Despite the loss of coverage, no significant loss in variant calling accuracy across the samples was observed and genetic diagnosis could be made with 100% concordance.

## Discussion

Diagnosis for LSDs is challenging due to several factors like phenotypic variability, the presence of overlapping clinical features across some LSDs, genetic heterogeneity and the difficulties associated with biochemical tests [2]. Recently, several studies have highlighted the incorporation of targeted NGS technologies as a potential diagnostic tool for LSDs [10, 11]. The essential advantage of using this approach includes unbiased interrogation of several genes at a time, thus enabling us to monitor a broader spectrum of diseases in a single test. This is especially beneficial in patients where the symptoms are not specific for a particular LSD and for LSDs where biochemical tests are not available. Fernandez-Marmiesse et al. for the first time demonstrated the use of a targeted sequencing assay to test 57 LSDs associated genes using in-solution capture as the enrichment method [10]. In the present study, we developed and applied a novel smMIP-based sequencing assay for the diagnosis of 29 common LSDs in India. We successfully demonstrated its ability to detect genetic abnormalities including both SNVs and CNVs by subjecting patient samples with previously identified genetic etiology and high clinical likelihood for one of the 29 common LSDs to the smMIP based assay study.

Despite a high proportion of targeted regions covered by the assay (99.2% of 53.7 kb), poor coverage was observed for genes (particularly *IDS, IDUA* and *GBA*) with low sequencing complexity or high sequence similarity with their pseudogene [33, 34]. This un-equivalency in target capture and sequencing of these genes is in congruence with observations made previously by Zanetti et al. [34]. For example, we observed poor diagnostic yield in clinically suspected MPS II cases. As exon 3 of the *IDS* gene is known to have a high sequence similarity with the pseudogene (*IDSP1*), no smMIP probes could be designed to capture this region with high specificity. Indeed, 4 patients received a genetic diagnosis of MPS II after Sanger sequencing was used to sequence exon 3 of the *IDS* gene in patients where the smMIP based assay didn’t identify causative variant. Like most NGS based assays (WES/WGS), one particular limitation of this assay is its inability to detect complex structural rearrangements. For example, smMIP-based assay cannot resolve and detect *IDSP1-*mediated *IDS* gene inversions or the RecNciI allele in the *GBA* gene, which is formed due to a non-homologous cross over between *GBA* and *GBAP1* genes. Indeed, recent guidelines for genetic testing of these genes recommend Sanger sequencing of poorly covered regions or regions with high sequence similarity with pseudogene [13]. Additionally, orthogonal methods such as PCR–RFLP HinfI assay are suggested to be used in the detection of *IDS*/*IDSP1* gene inversions in genetically undiagnosed MPS II patients as mentioned previously [35].

Importantly, the smMIP-based assay has high sensitivity and specificity for detection of both SNVs and CNVs due to the availability of UMBs in the backbone of the smMIP probes. This is reflected in the 98% and 100% concordance in SNV and CNV calling in the validation cohort. Furthermore, assessment of the assay’s diagnostic yield in a cohort of 300 patient samples ranged from 2.4 to 83%. This large variance in diagnostic yield is due to the heterogeneity of the patient cohort which consisted of 187 patient samples which had previously been diagnosed LSD using biochemical assay, 72 patient samples with a high clinical likelihood of LSD and 41 patient samples with a low clinical likelihood of LSD. Indeed, patients with prior biochemical assay based diagnosis or a high clinical suspicion of LSD showed a remarkable diagnostic yield of 83% and 74%, respectively, in comparison of the low clinical suspicion group, which showed yield of only 2.4%. This likely signifies and further emphasizes requirement of a deep clinical phenotyping before the used of NGS based assays in order to receive high diagnostic yields. Of note, observed diagnostic yields in both biochemically confirmed cases and high clinical suspicion cases are significantly higher than the yield of 62% in biochemically confirmed cases by Zanetti et al. [34]. The observed yields are also higher than that the reported yield of 67% by Di Fruscio et al. using Lysoplex in a group of 48 NCL patients [9] and 30% yield obtained using WES on 14 patients with an LSD suspicion reported by Wang et al. 2017 [36]. The higher diagnostic yield achieved in the high-index cohort in the present study is likely because of the deep clinical characterization of the patients before referring them for NGS-based panel studies. Interestingly, the type of disease for which a gene panel is offered also influences its diagnostic yield. For instance, the yield for a hypertrophic cardiomyopathy panel is as high as 32% [37] but for a congenital glycosylation disorders gene panel, it is only 14.8% [38]. This suggests that the complexity of the disease nature in question and its clinical presentation dictate the diagnostic success of gene panels. LSDs, in general, may present with a more specific phenotype. This explains the variability in diagnostic yield reported by different NGS panel studies for LSDs. It ranges from 15% reported by Gheldhof et al. in a cohort of 150 cases to 40% reported by Fernandez-Marmiesse et al. 2014 in a group of 66 suspected LSD patients [10, 39]. In addition, the smMIP-based assay could detect multi-exon and single-exon deletions in eight cases (~ 5%) of the total diagnosed cases. Large deletions in ~ 3–5% of cases of MPS II, Krabbe, and Niemann-pick diseases have been observed previously in the literature [40–42]. This observation is further strengthened by the in-ability to further improve diagnostic yield for 29 targeted LSDs using WES or WGS in patients where the smMIP based assay didn’t detect a causative variant.

An interesting observation was made for 3 biochemically diagnosed cases where the smMIP based assay corrected previous misdiagnoses. In two cases, biochemical diagnosis suggested Gaucher's disease, however, the smMIP based assay identified a causative variant in the *NPC1* gene. Previously, it has been known that Niemann Pick type C is a differential diagnosis for Gaucher disease and is associated with falsely low beta-glucosidase activity [43]. Likewise, for another case with a reduced activity of alpha-iduronidase enzyme activity, a diagnosis of MPS I was made. However, the smMIP-based assay detected a variant in the *GNPTAB* gene. Patients with a defect in the *GNPTAB* gene display reduced activity of multiple lysosomal enzymes as there is a defect in the enzyme GlcNAc-1-phosphotransferase [44]. This enzyme is critical for tagging mannose-6-phosphate (M6P) to lysosomal enzymes so that they can bind to the M6P receptors on the trans-Golgi network [44]. Hence, patients with Mucoplipidosis-II/III can easily be misdiagnosed as MPS cases. Thus, genetic testing following biochemical testing is critical in such cases and the above observations highlight the strength of the assay in providing a diagnosis in cases with clinical heterogeneity. Previously, using this assay, we could also identify MLD due to activator protein deficiency in an adult patient [45], which could have been missed by a biochemical assay as these patients show normal levels of the arylsulfatase-A enzyme activity (Table 1). Thus, the assay can aid in the diagnosis of diseases like Niemann-Pick type C1/C2, saposin A/B/C deficiency as well as neuronal ceroid lipofuscinosis type 6, for which there are no well-established biochemical diagnostic tests available (Table 1).

Lastly, comparative analysis of the sequencing quality between DNA extracted from whole blood and DBS sample suggests potential utility of the smMIP based assay for newborn screening programs for detection of common LSDs in a given population. However, unlike whole blood samples, further evaluation and optimisation of the assay parameters for DBS samples may be warranted before its utilization in a clinical setting.

The costs of our smMIP-based assay are relatively low compared to the currently employed diagnostic pathway consisting of biochemical testing for LSD diagnosis. Although smMIPs require a relatively high initial investment, the per-patient library preparation and sequencing cost is estimated to be as low as US$73 on the Illumina MiSeq platform with Micro v2 flowcell and 200 × average sequence depth. This equates to approximately US$3.2 per gene per sample tested. However, in order to draw a definitive conclusion, further evaluation of cost-effective analysis needs to performed by comparing it with the costs incurred using the existing diagnostic route as well as calculating the time taken to reach to a diagnosis. Nonetheless, with the combined ability to detect both SNVs and CNVs, ease of use, high diagnostic yield and low costs, the utility of smMIP-based assay for 29 common LSDs irrespective of the clinical phenotype, especially in low-middle income countries, may allow for a paradigm shift in the clinical diagnostic pathway. Due to these advantages, clinical implementation of smMIP based NGS assays have previously been carried out in somatic microsatellite instability testing in colorectal cancer [46] and germline *BRCA* gene testing for identification of patients with hereditary breast and ovarian cancer [47].

## Conclusions

We describe a novel and cost-efficient assay for genetic diagnosis of 29 common LSDs. We have shown that the assay can detect both SNVs and CNVs, and can be applied on DNA extracted from whole blood and DBS samples. The assay has proved to a powerful addition to the current diagnostic assay repertoire, and both patients and doctors can benefit greatly from utilizing this technique, especially in resource-limited settings.

### Supplementary Information

**Additional file 1**. List of study sites and recruited patients.

**Additional file 2**. Single molecule molecular inversion probe sequences of 903 probes along with their target enrichment site coordinates used in the smMIP based assay.

**Additional file 3**. Overall distribution of reads across the 903 smMIP molecules before and after rebalancing the smMIP pool. Considering, total 903 probes, we expect 0.001 proportion of reads to be the optimum value by each probe. The navy blue denotes the optimum proportion of read value i.e 0.001. A lower cut-off value of 0.0001 and higher cut-off value of 0.01 was set as the optimum range to assess the efficiency of the probes.

**Additional file 4**. Clinical data and biochemical assay results of 187 patients with prior biochemical diagnosis of LSD in the diagnostic yield cohort.

**Additional file 5**. Clinical data and smMIP based assay result in 113 patients with a clinical suspicion of LSD in the diagnostic yield cohort.

**Additional file 6**. Comparative data for Dried Blood Spot (DBS) extracted DNA and Manual blood extracted DNA used in smMIP assay.

**Additional file 7**. Coverage data of 903 probes using Dried Blood Spot (DBS) extracted DNA and Manual blood extracted DNA in smMIP-NGS assay.

## Abbreviations

LSDs: Lysosomal storage disorders
smMIPs: Single-molecule molecular inversion probes
ERT: Enzyme replacement therapy
NGS: Next generation sequencing
MLPA: Multiplex ligation probe dependent amplification
CNV: Copy number variations
SNV: Single nucleotide variants
UMB: Unique molecular barcode
LMICs: Low-middle income countries
ACMG: American College of Medical Genetics
DBS: Dried blood spot
WES: Whole exome sequencing
WGS: Whole genome sequencing
