# Supplementary figures and images for: Development, validation and application of single molecule molecular inversion probe based novel integrated genetic screening method for 29 common lysosomal storage disorders in India
Source: Hum Genomics. 2024 May 10;18:46. doi: 10.1186/s40246-024-00613-9 (PMC11088154; doi:10.1186/s40246-024-00613-9)

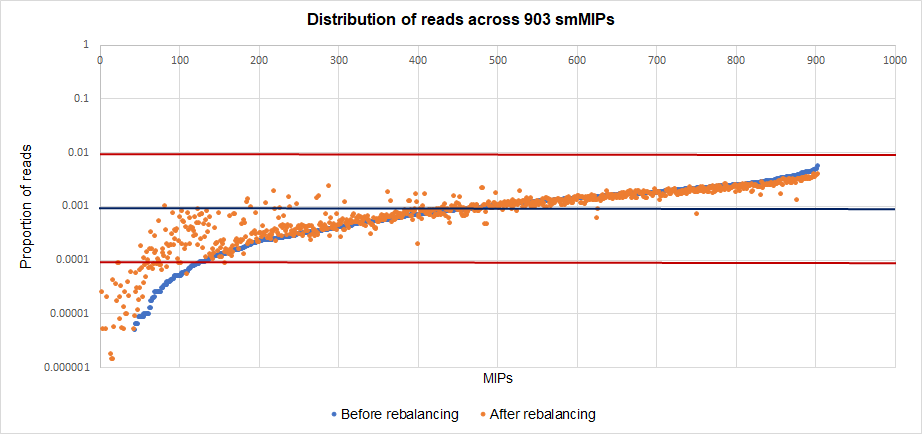

Supplement: Supplementary file 3 — Additional file 3. Overall distribution of reads across the 903 smMIP molecules before and after rebalancing the smMIP pool. Considering, total 903 probes, we expect 0.001 proportion of reads to be the optimum value by each probe. The navy blue denotes the optimum proportion of read value i.e 0.001. A lower cut-off value of 0.0001 and higher cut-off value of 0.01 was set as the optimum range to assess the efficiency of the probes. [file 40246_2024_613_MOESM3_ESM.tif]
