# Supplementary material for: Development, validation and application of single molecule molecular inversion probe based novel integrated genetic screening method for 29 common lysosomal storage disorders in India
Source: Hum Genomics. 2024 May 10;18:46. doi: 10.1186/s40246-024-00613-9 (PMC11088154; doi:10.1186/s40246-024-00613-9)
Supplement: Supplementary file 6 — Additional file 6. Comparative data for Dried Blood Spot (DBS) extracted DNA and Manual blood extracted DNA used in smMIP assay. [file 40246_2024_613_MOESM6_ESM.pptx]

## Slide 1
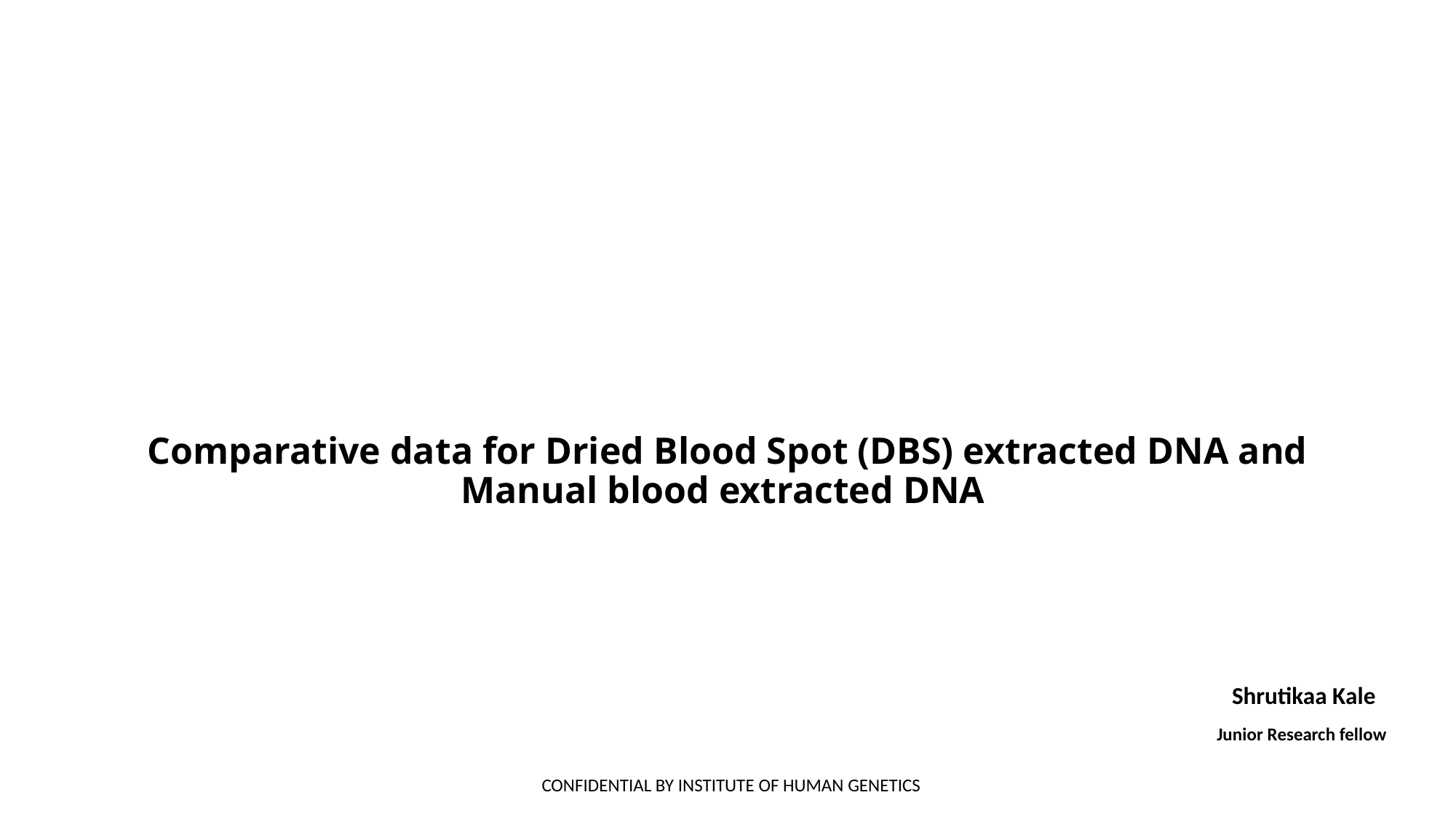

# Comparative data for Dried Blood Spot (DBS) extracted DNA and Manual blood extracted DNA
Shrutikaa Kale
Junior Research fellow

## Slide 2
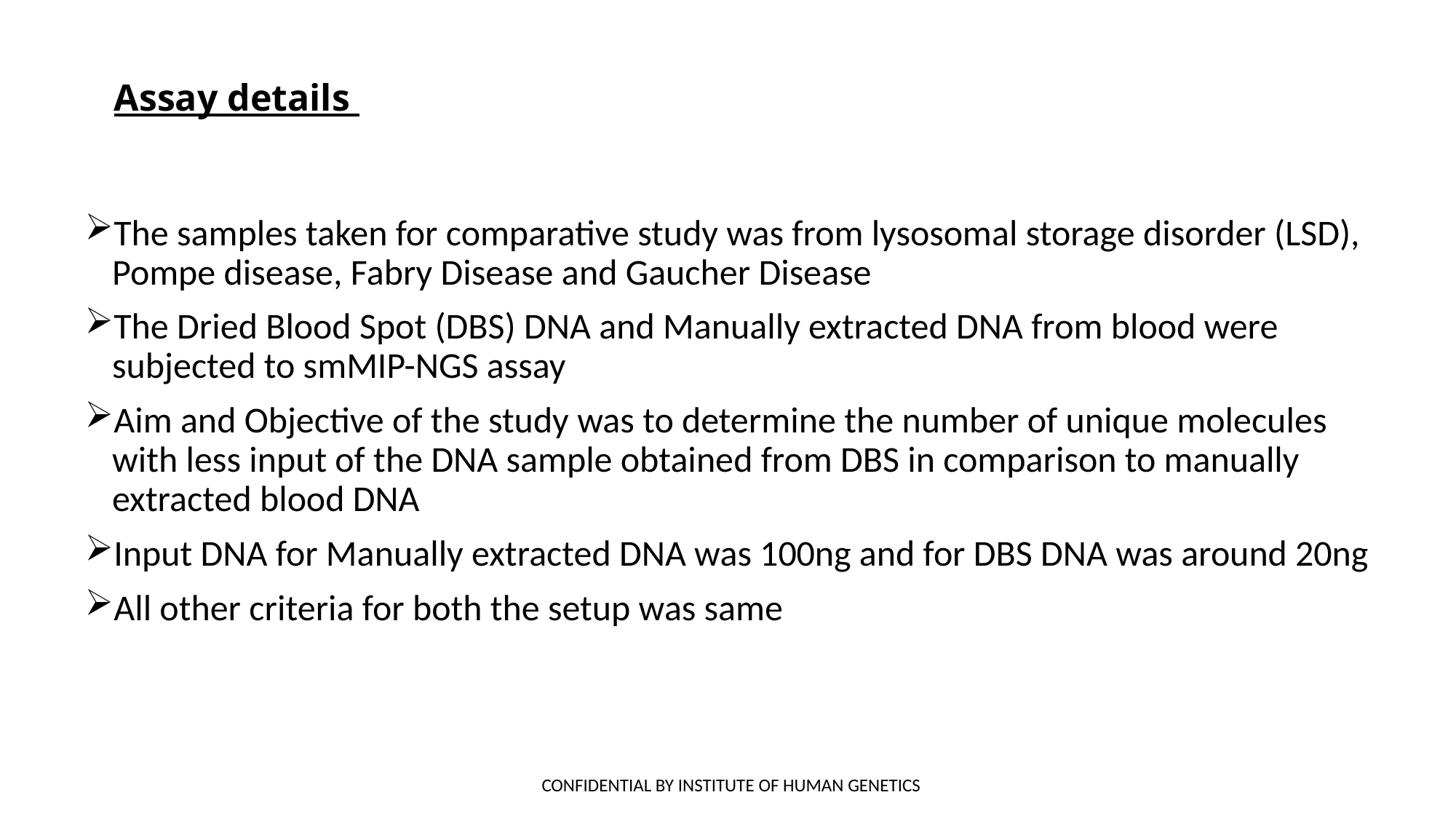

# Assay details
The samples taken for comparative study was from lysosomal storage disorder (LSD), Pompe disease, Fabry Disease and Gaucher Disease
The Dried Blood Spot (DBS) DNA and Manually extracted DNA from blood were subjected to smMIP-NGS assay
Aim and Objective of the study was to determine the number of unique molecules with less input of the DNA sample obtained from DBS in comparison to manually extracted blood DNA
Input DNA for Manually extracted DNA was 100ng and for DBS DNA was around 20ng
All other criteria for both the setup was same

## Slide 3
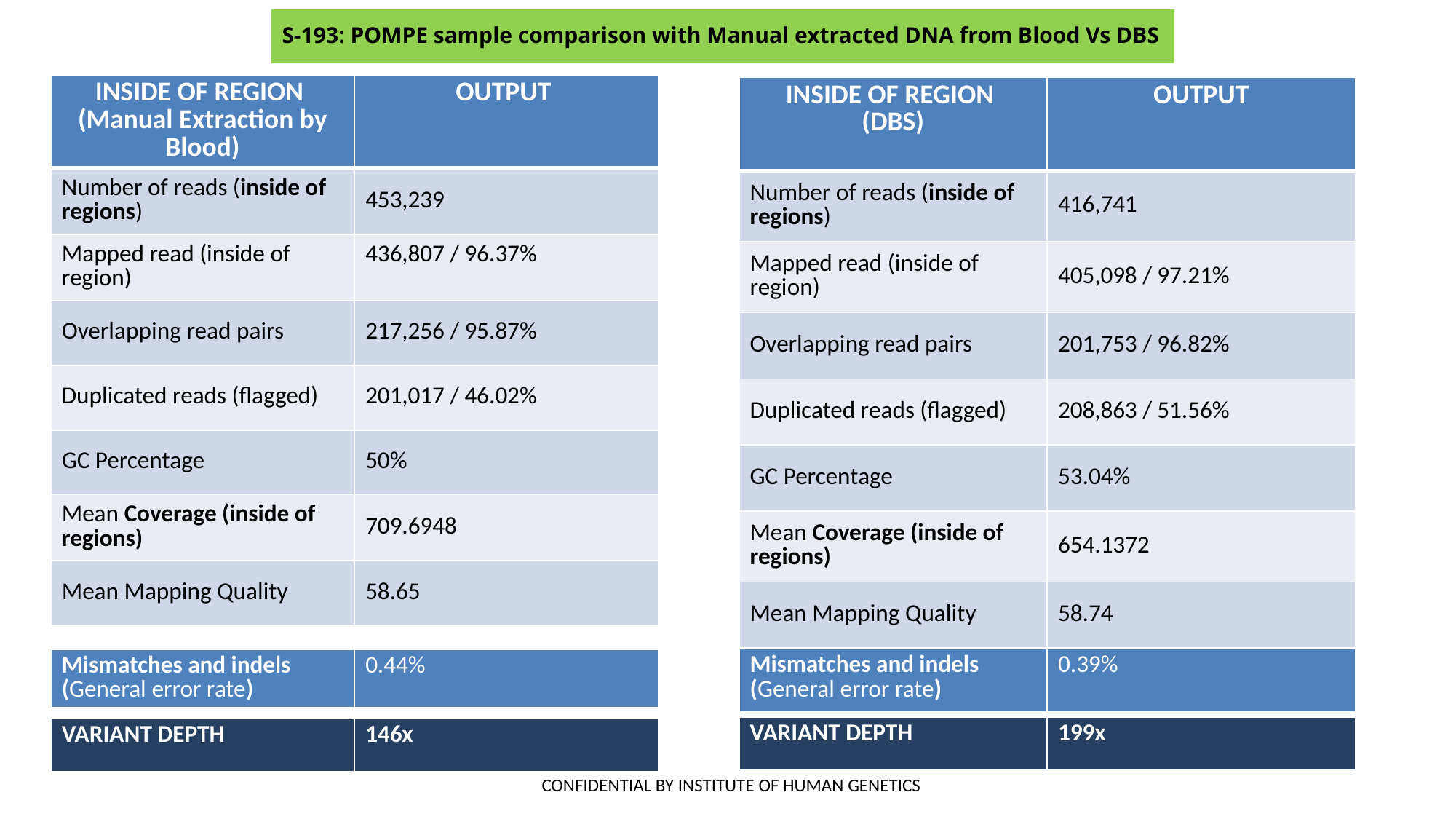

# S-193: POMPE sample comparison with Manual extracted DNA from Blood Vs DBS
| INSIDE OF REGION (Manual Extraction by Blood) | OUTPUT |
| --- | --- |
| Number of reads (inside of regions) | 453,239 |
| Mapped read (inside of region) | 436,807 / 96.37% |
| Overlapping read pairs | 217,256 / 95.87% |
| Duplicated reads (flagged) | 201,017 / 46.02% |
| GC Percentage | 50% |
| Mean Coverage (inside of regions) | 709.6948 |
| Mean Mapping Quality | 58.65 |
| INSIDE OF REGION (DBS) | OUTPUT |
| --- | --- |
| Number of reads (inside of regions) | 416,741 |
| Mapped read (inside of region) | 405,098 / 97.21% |
| Overlapping read pairs | 201,753 / 96.82% |
| Duplicated reads (flagged) | 208,863 / 51.56% |
| GC Percentage | 53.04% |
| Mean Coverage (inside of regions) | 654.1372 |
| Mean Mapping Quality | 58.74 |
| Mismatches and indels (General error rate) | 0.39% |
| --- | --- |
| Mismatches and indels (General error rate) | 0.44% |
| --- | --- |
| VARIANT DEPTH | 199x |
| --- | --- |
| VARIANT DEPTH | 146x |
| --- | --- |

## Slide 4
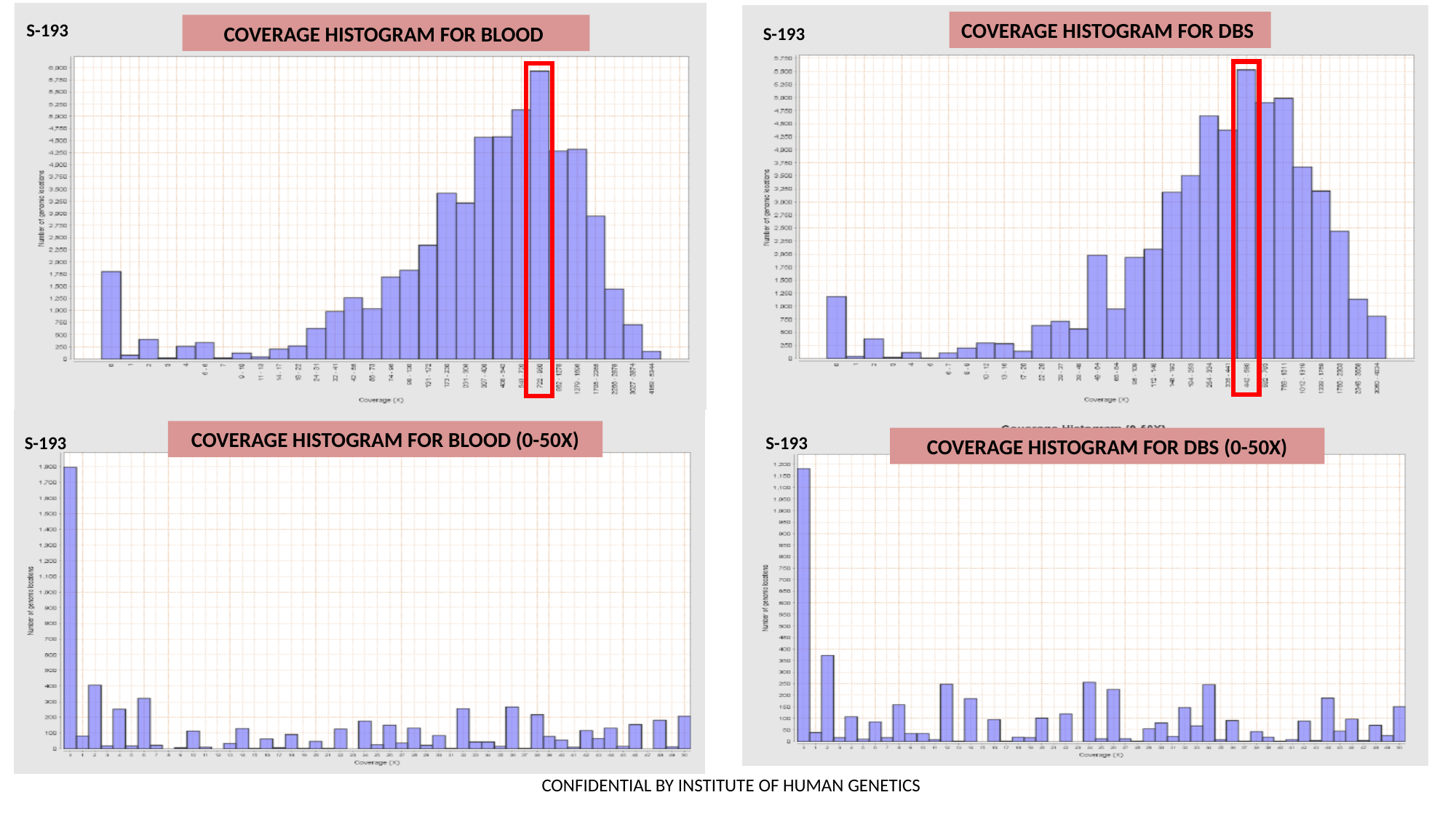

COVERAGE HISTOGRAM FOR DBS
S-193
COVERAGE HISTOGRAM FOR BLOOD
S-193
COVERAGE HISTOGRAM FOR BLOOD (0-50X)
S-193
S-193
COVERAGE HISTOGRAM FOR DBS (0-50X)

## Slide 5
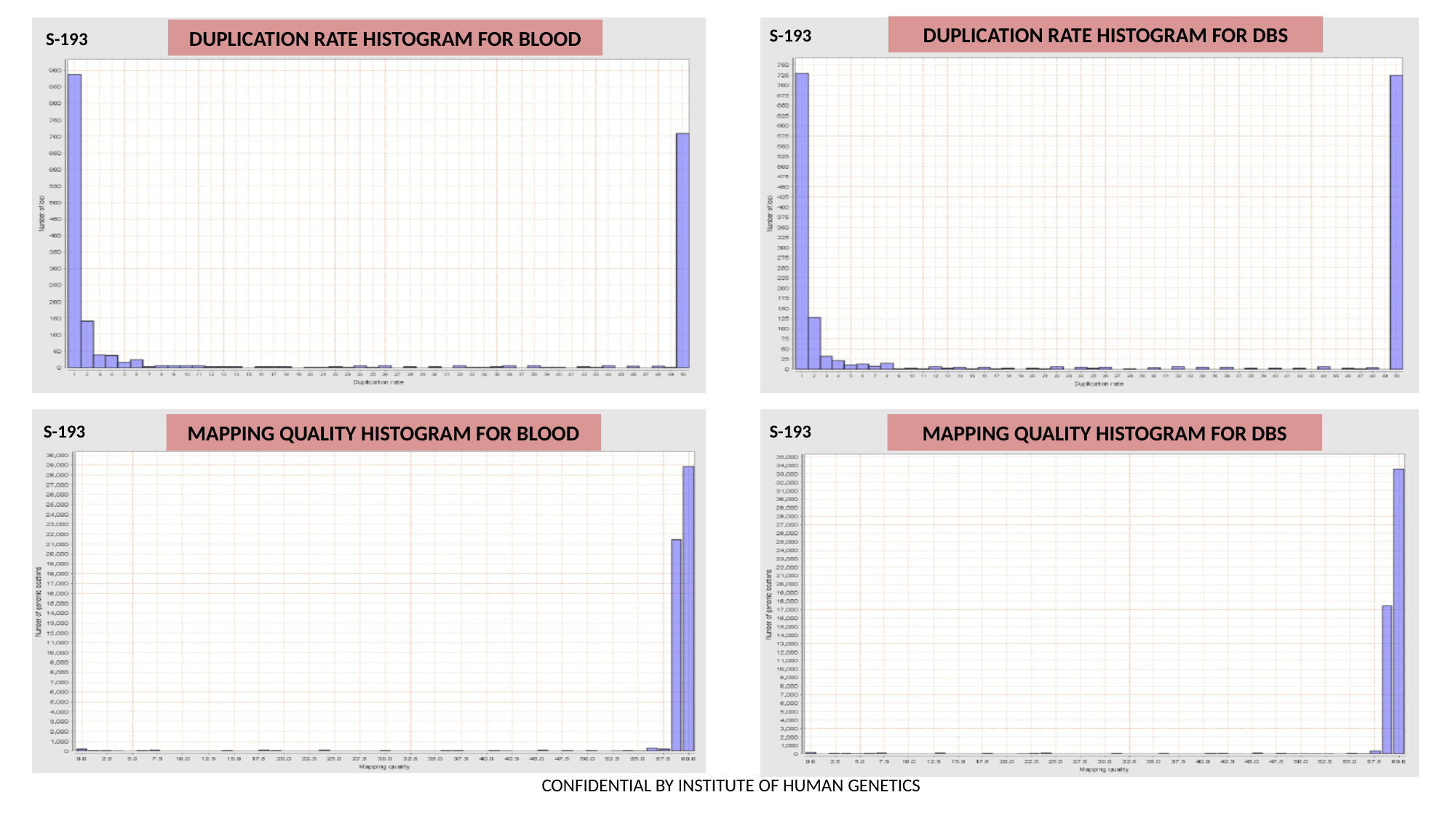

DUPLICATION RATE HISTOGRAM FOR DBS
S-193
DUPLICATION RATE HISTOGRAM FOR BLOOD
S-193
S-193
MAPPING QUALITY HISTOGRAM FOR BLOOD
MAPPING QUALITY HISTOGRAM FOR DBS
S-193

## Slide 6
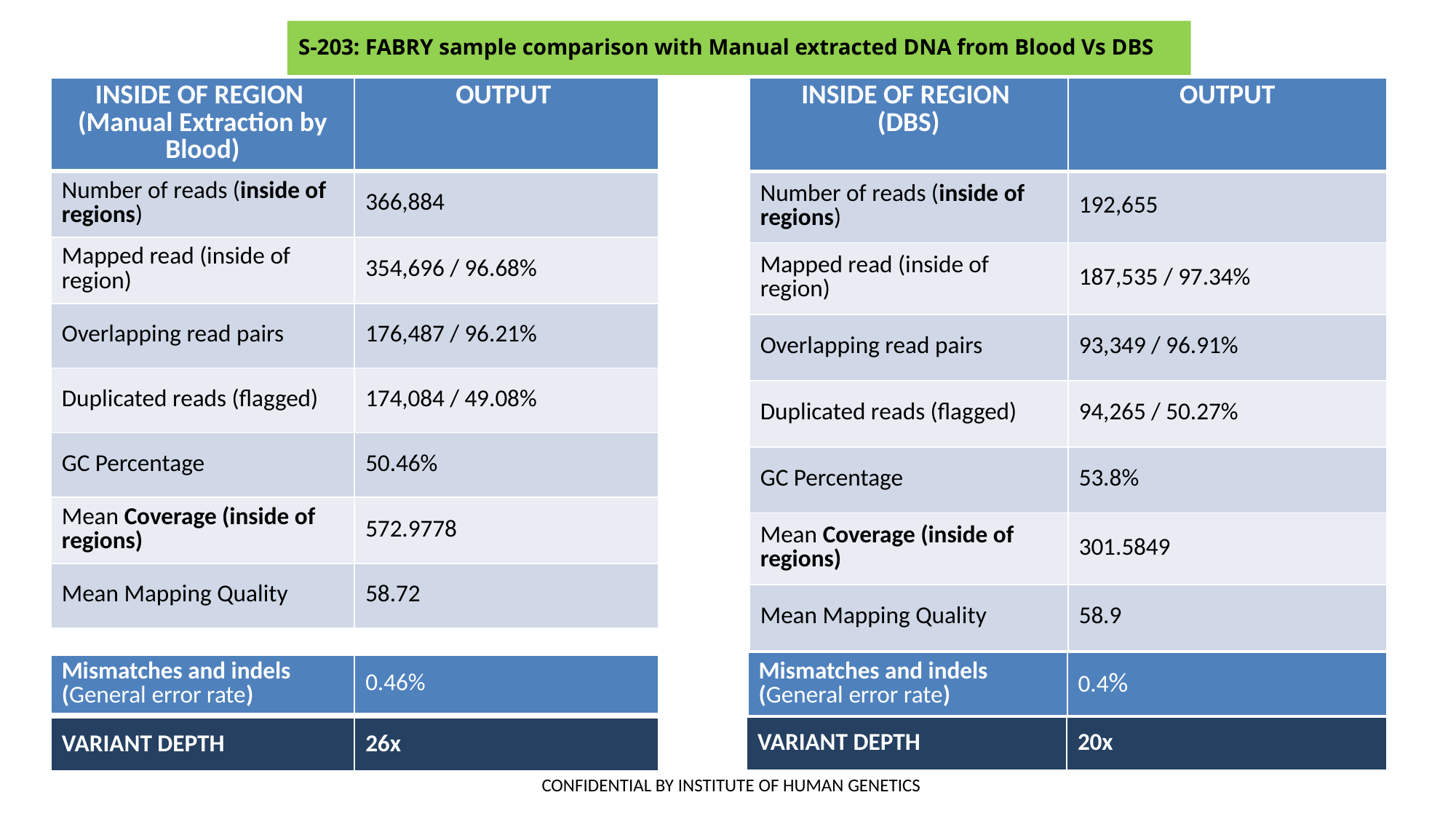

# S-203: FABRY sample comparison with Manual extracted DNA from Blood Vs DBS
| INSIDE OF REGION (Manual Extraction by Blood) | OUTPUT |
| --- | --- |
| Number of reads (inside of regions) | 366,884 |
| Mapped read (inside of region) | 354,696 / 96.68% |
| Overlapping read pairs | 176,487 / 96.21% |
| Duplicated reads (flagged) | 174,084 / 49.08% |
| GC Percentage | 50.46% |
| Mean Coverage (inside of regions) | 572.9778 |
| Mean Mapping Quality | 58.72 |
| INSIDE OF REGION (DBS) | OUTPUT |
| --- | --- |
| Number of reads (inside of regions) | 192,655 |
| Mapped read (inside of region) | 187,535 / 97.34% |
| Overlapping read pairs | 93,349 / 96.91% |
| Duplicated reads (flagged) | 94,265 / 50.27% |
| GC Percentage | 53.8% |
| Mean Coverage (inside of regions) | 301.5849 |
| Mean Mapping Quality | 58.9 |
| Mismatches and indels (General error rate) | 0.4% |
| --- | --- |
| Mismatches and indels (General error rate) | 0.46% |
| --- | --- |
| VARIANT DEPTH | 20x |
| --- | --- |
| VARIANT DEPTH | 26x |
| --- | --- |

## Slide 7
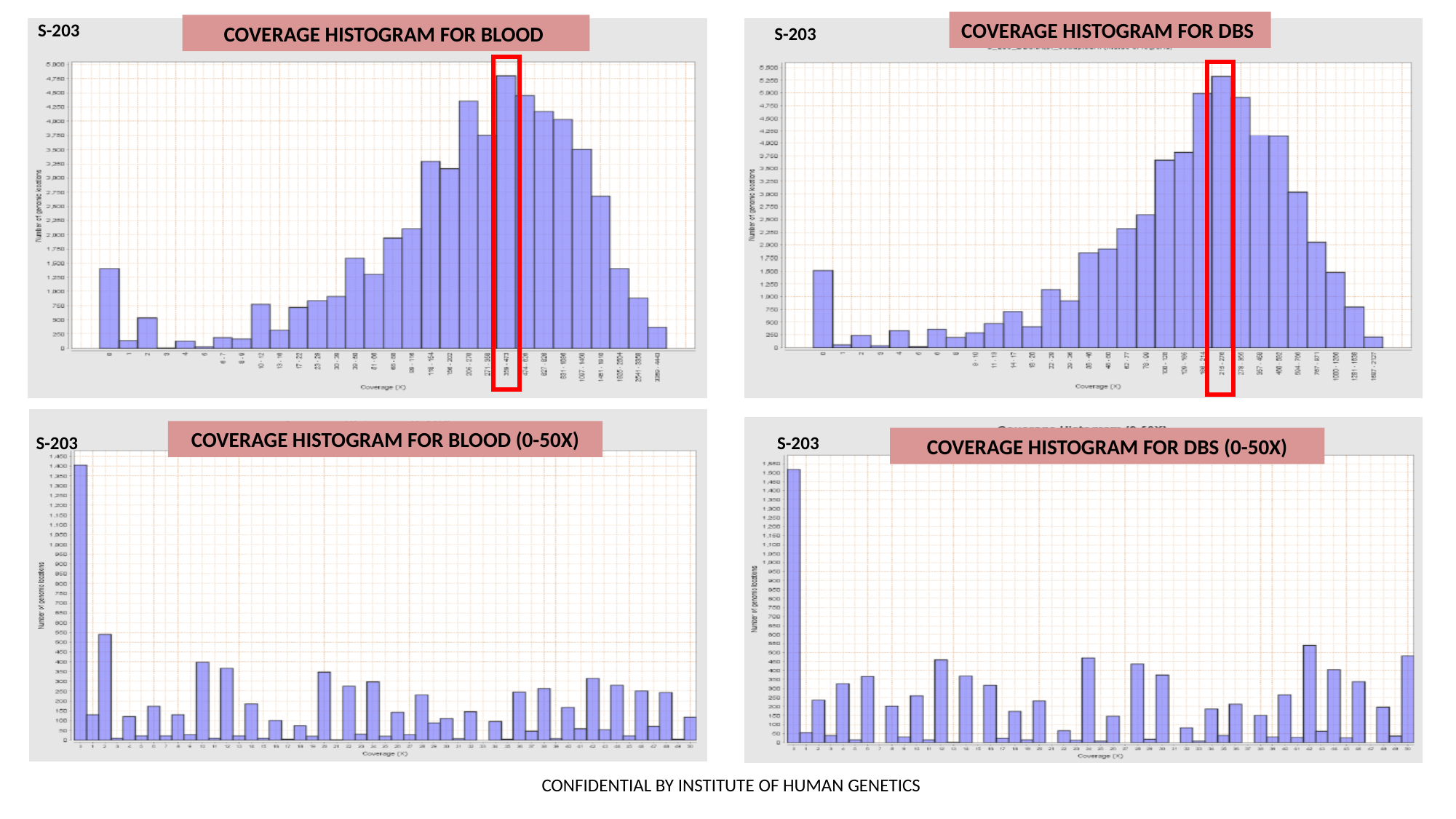

COVERAGE HISTOGRAM FOR DBS
S-203
COVERAGE HISTOGRAM FOR BLOOD
S-203
COVERAGE HISTOGRAM FOR BLOOD (0-50X)
S-203
S-203
COVERAGE HISTOGRAM FOR DBS (0-50X)

## Slide 8
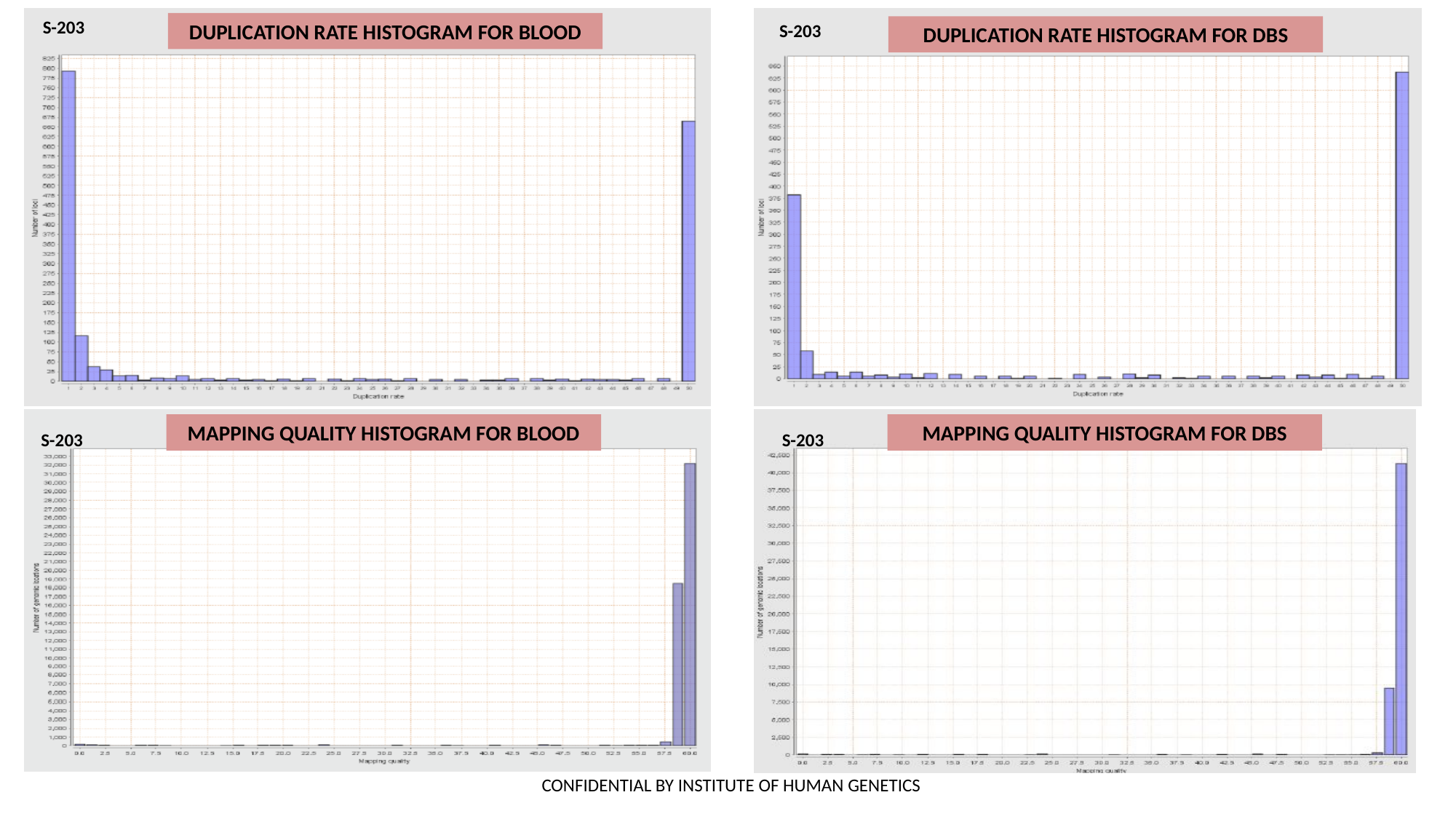

S-203
DUPLICATION RATE HISTOGRAM FOR BLOOD
S-203
DUPLICATION RATE HISTOGRAM FOR DBS
MAPPING QUALITY HISTOGRAM FOR BLOOD
MAPPING QUALITY HISTOGRAM FOR DBS
S-203
S-203

## Slide 9
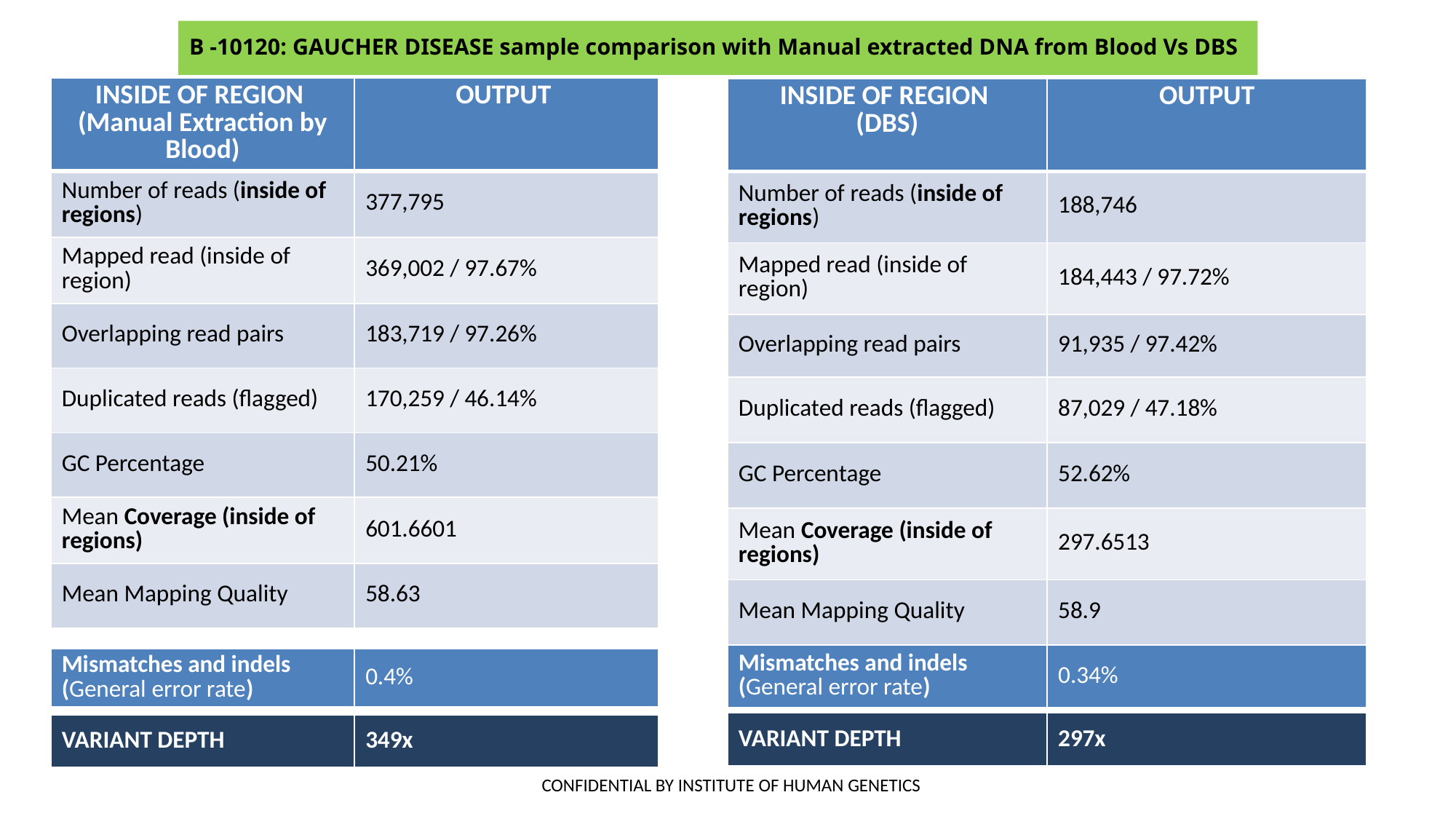

# B -10120: GAUCHER DISEASE sample comparison with Manual extracted DNA from Blood Vs DBS
| INSIDE OF REGION (Manual Extraction by Blood) | OUTPUT |
| --- | --- |
| Number of reads (inside of regions) | 377,795 |
| Mapped read (inside of region) | 369,002 / 97.67% |
| Overlapping read pairs | 183,719 / 97.26% |
| Duplicated reads (flagged) | 170,259 / 46.14% |
| GC Percentage | 50.21% |
| Mean Coverage (inside of regions) | 601.6601 |
| Mean Mapping Quality | 58.63 |
| INSIDE OF REGION (DBS) | OUTPUT |
| --- | --- |
| Number of reads (inside of regions) | 188,746 |
| Mapped read (inside of region) | 184,443 / 97.72% |
| Overlapping read pairs | 91,935 / 97.42% |
| Duplicated reads (flagged) | 87,029 / 47.18% |
| GC Percentage | 52.62% |
| Mean Coverage (inside of regions) | 297.6513 |
| Mean Mapping Quality | 58.9 |
| Mismatches and indels (General error rate) | 0.34% |
| --- | --- |
| Mismatches and indels (General error rate) | 0.4% |
| --- | --- |
| VARIANT DEPTH | 297x |
| --- | --- |
| VARIANT DEPTH | 349x |
| --- | --- |

## Slide 10
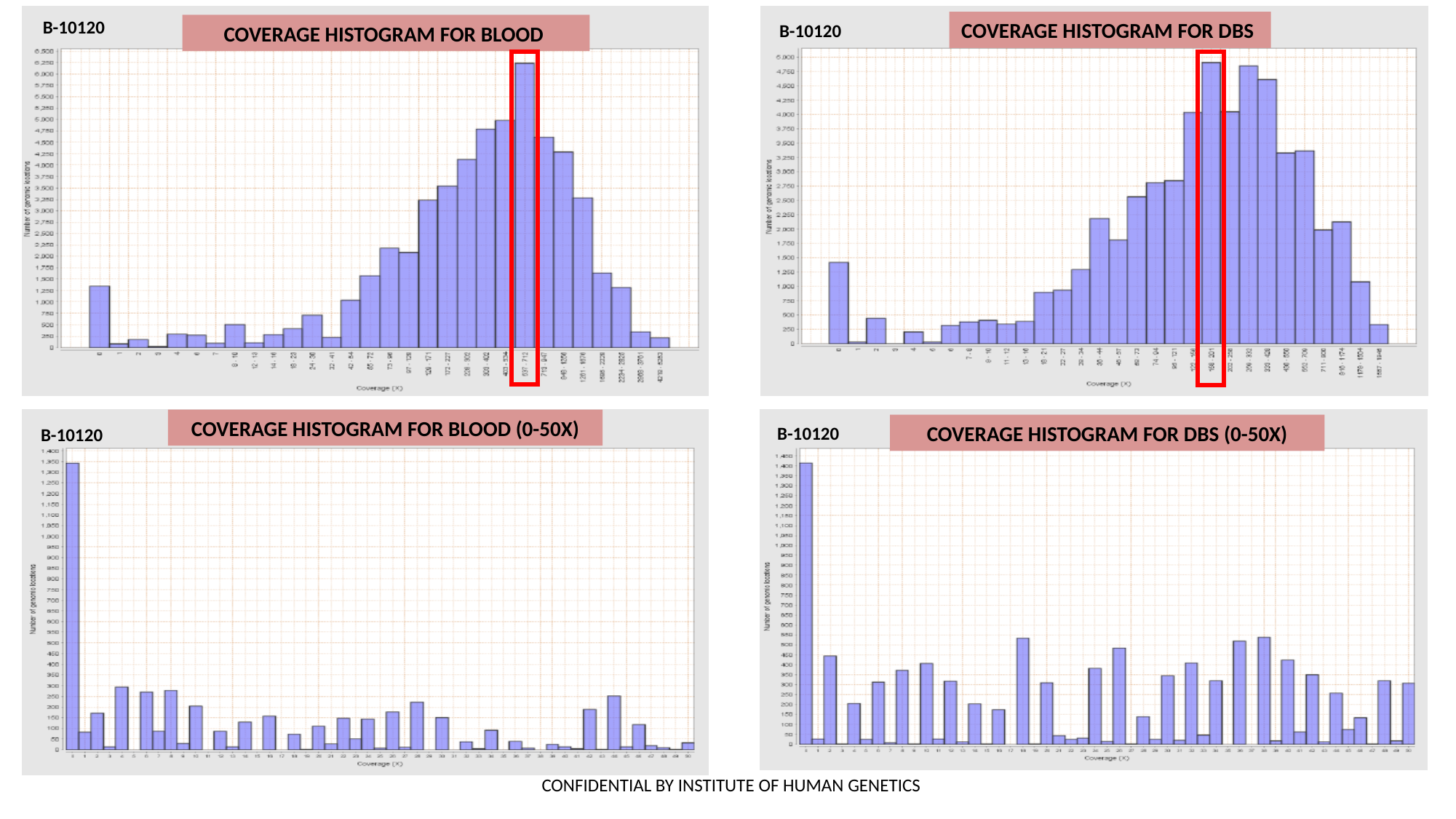

B-10120
COVERAGE HISTOGRAM FOR DBS
B-10120
COVERAGE HISTOGRAM FOR BLOOD
COVERAGE HISTOGRAM FOR BLOOD (0-50X)
COVERAGE HISTOGRAM FOR DBS (0-50X)
B-10120
B-10120

## Slide 11
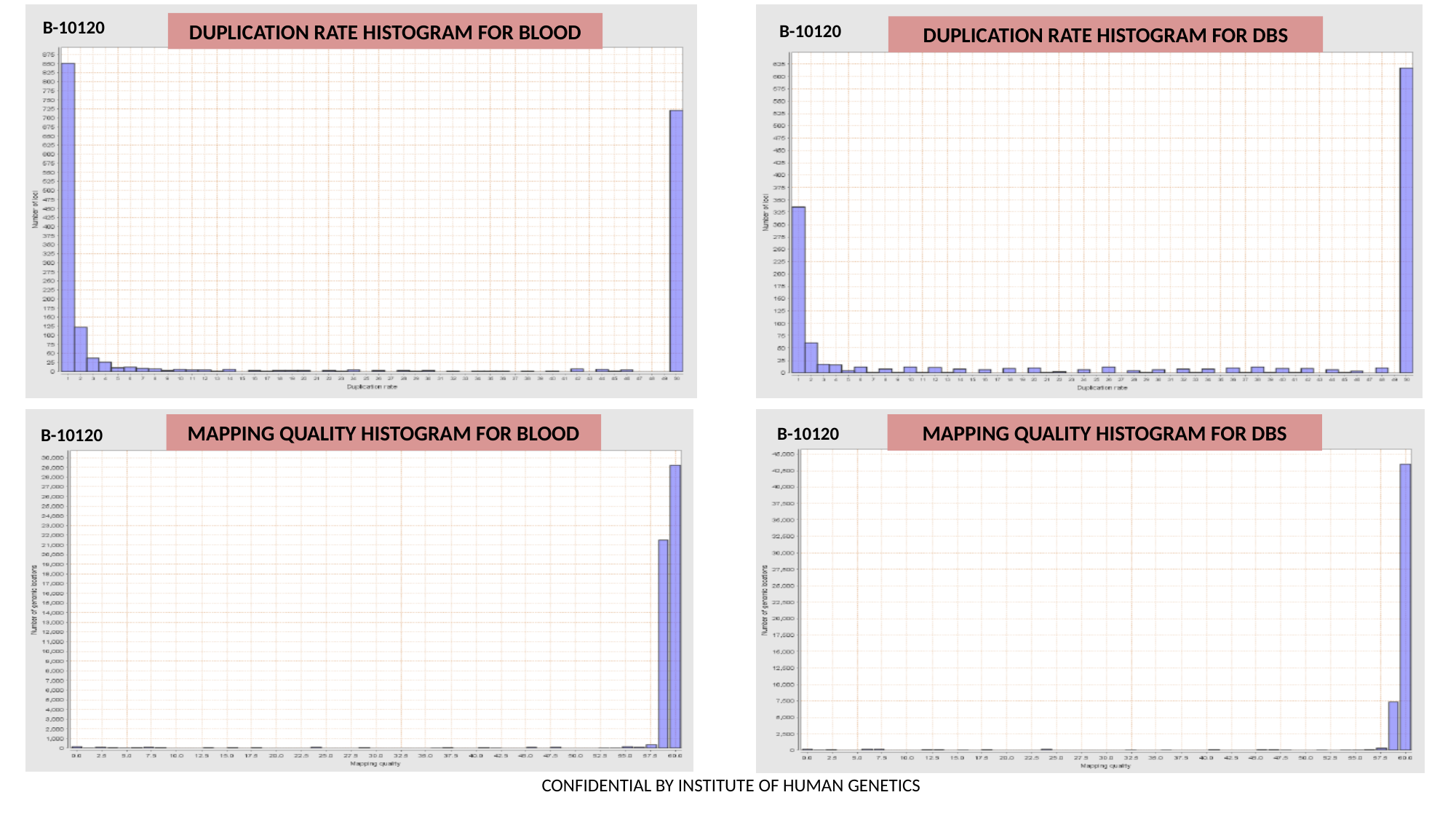

B-10120
DUPLICATION RATE HISTOGRAM FOR BLOOD
B-10120
DUPLICATION RATE HISTOGRAM FOR DBS
MAPPING QUALITY HISTOGRAM FOR BLOOD
MAPPING QUALITY HISTOGRAM FOR DBS
B-10120
B-10120

## Slide 12
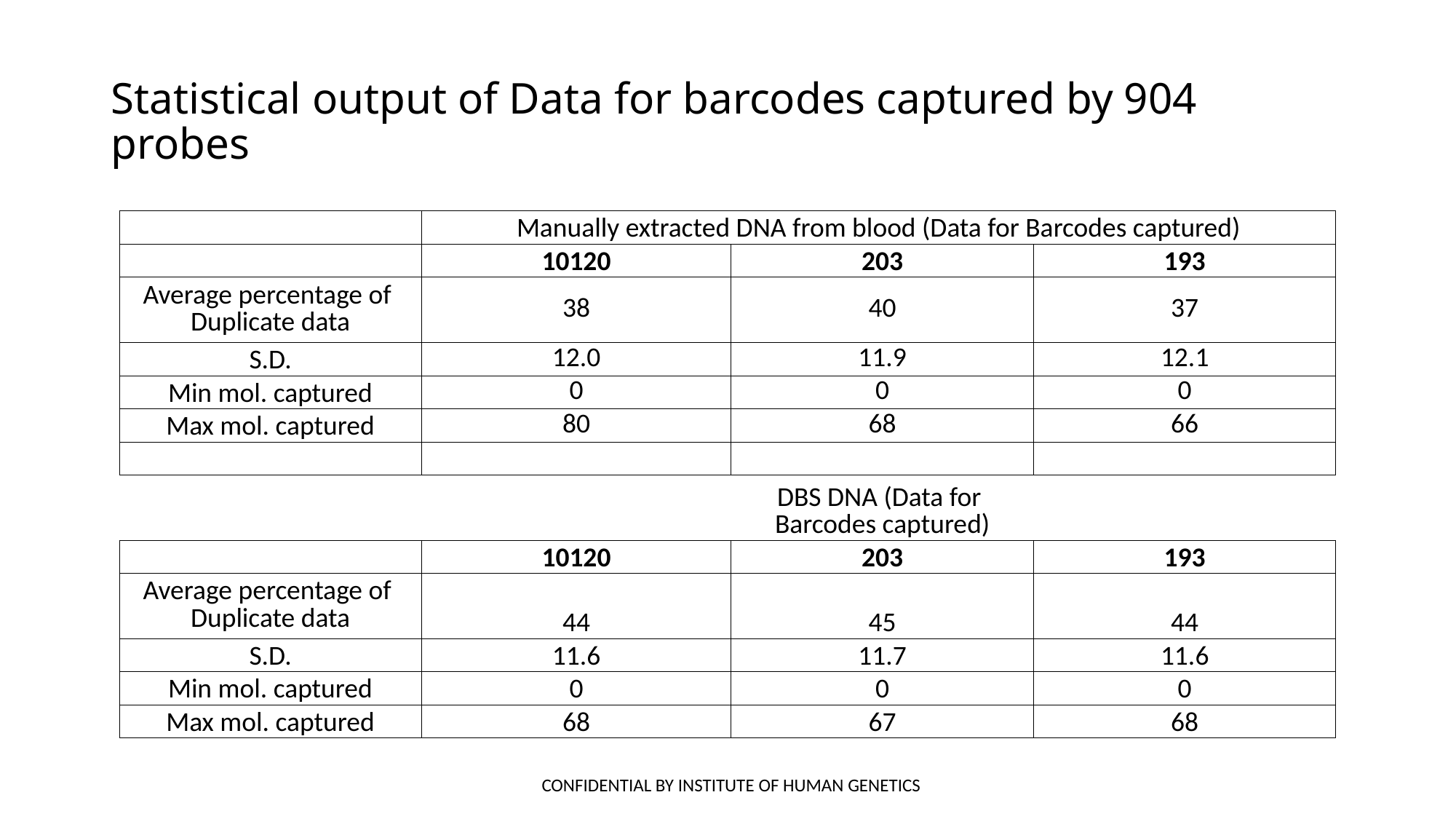

# Statistical output of Data for barcodes captured by 904 probes
| | Manually extracted DNA from blood (Data for Barcodes captured) | | |
| --- | --- | --- | --- |
| | 10120 | 203 | 193 |
| Average percentage of Duplicate data | 38 | 40 | 37 |
| S.D. | 12.0 | 11.9 | 12.1 |
| Min mol. captured | 0 | 0 | 0 |
| Max mol. captured | 80 | 68 | 66 |
| | | | |
| | | DBS DNA (Data for Barcodes captured) | |
| | 10120 | 203 | 193 |
| Average percentage of Duplicate data | 44 | 45 | 44 |
| S.D. | 11.6 | 11.7 | 11.6 |
| Min mol. captured | 0 | 0 | 0 |
| Max mol. captured | 68 | 67 | 68 |

## Slide 13
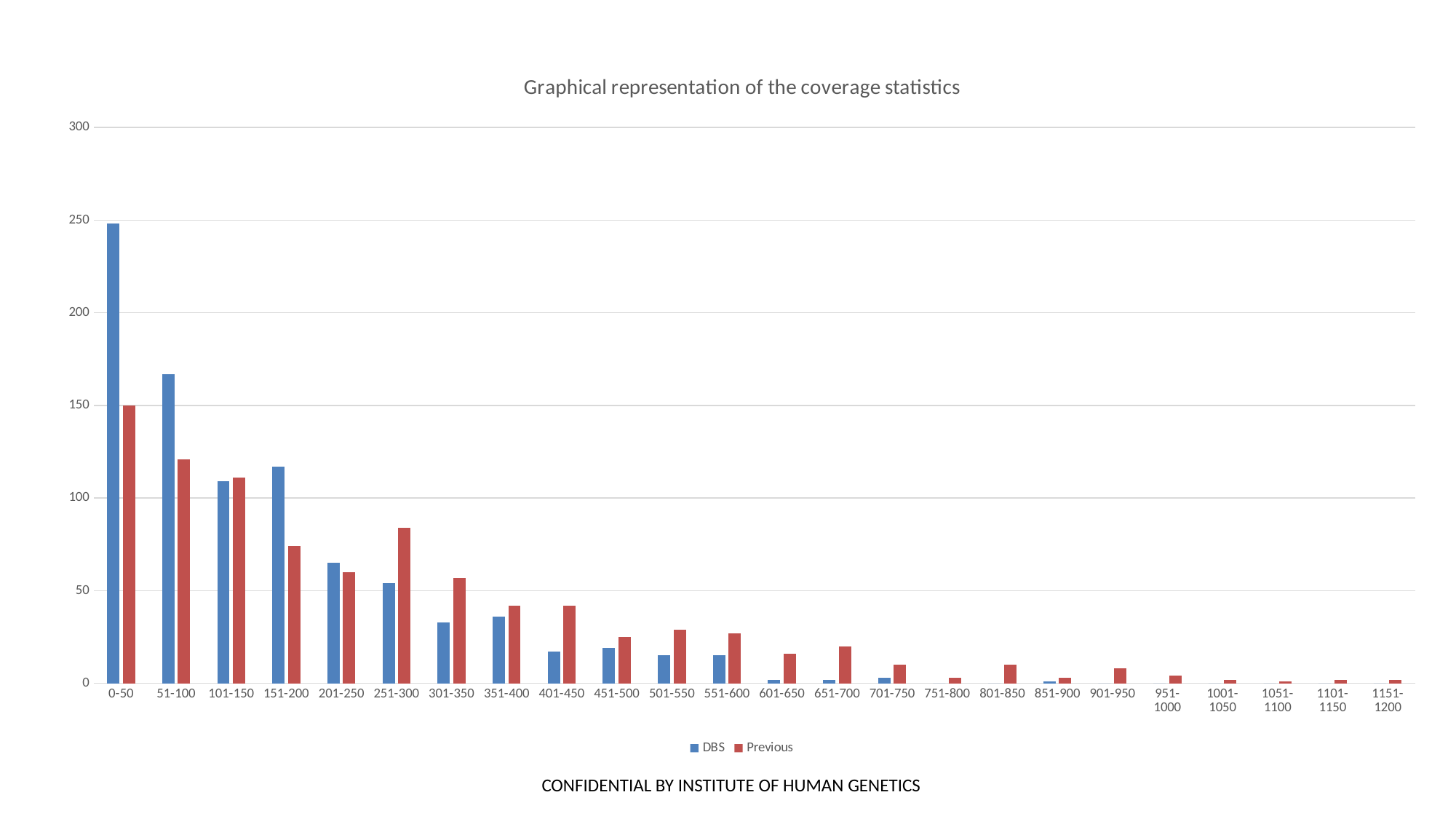

### Chart:  Graphical representation of the coverage statistics
| Category | DBS | Previous |
|---|---|---|
| 0-50 | 248.0 | 150.0 |
| 51-100 | 167.0 | 121.0 |
| 101-150 | 109.0 | 111.0 |
| 151-200 | 117.0 | 74.0 |
| 201-250 | 65.0 | 60.0 |
| 251-300 | 54.0 | 84.0 |
| 301-350 | 33.0 | 57.0 |
| 351-400 | 36.0 | 42.0 |
| 401-450 | 17.0 | 42.0 |
| 451-500 | 19.0 | 25.0 |
| 501-550 | 15.0 | 29.0 |
| 551-600 | 15.0 | 27.0 |
| 601-650 | 2.0 | 16.0 |
| 651-700 | 2.0 | 20.0 |
| 701-750 | 3.0 | 10.0 |
| 751-800 | 0.0 | 3.0 |
| 801-850 | 0.0 | 10.0 |
| 851-900 | 1.0 | 3.0 |
| 901-950 | 0.0 | 8.0 |
| 951-1000 | 0.0 | 4.0 |
| 1001-1050 | 0.0 | 2.0 |
| 1051-1100 | 0.0 | 1.0 |
| 1101-1150 | 0.0 | 2.0 |
| 1151-1200 | 0.0 | 2.0 |

## Slide 14
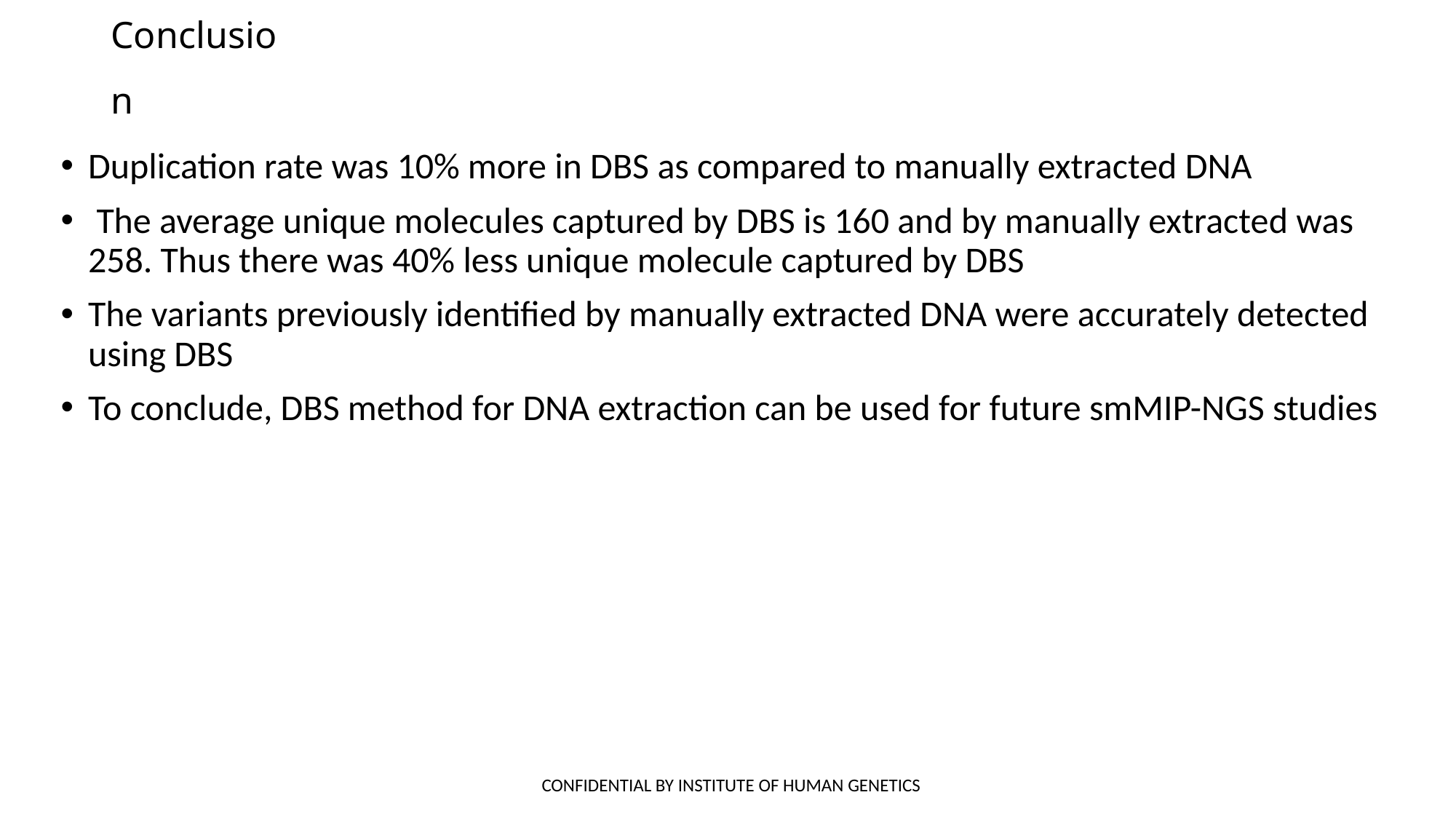

# Conclusion
Duplication rate was 10% more in DBS as compared to manually extracted DNA
 The average unique molecules captured by DBS is 160 and by manually extracted was 258. Thus there was 40% less unique molecule captured by DBS
The variants previously identified by manually extracted DNA were accurately detected using DBS
To conclude, DBS method for DNA extraction can be used for future smMIP-NGS studies

## Slide 15
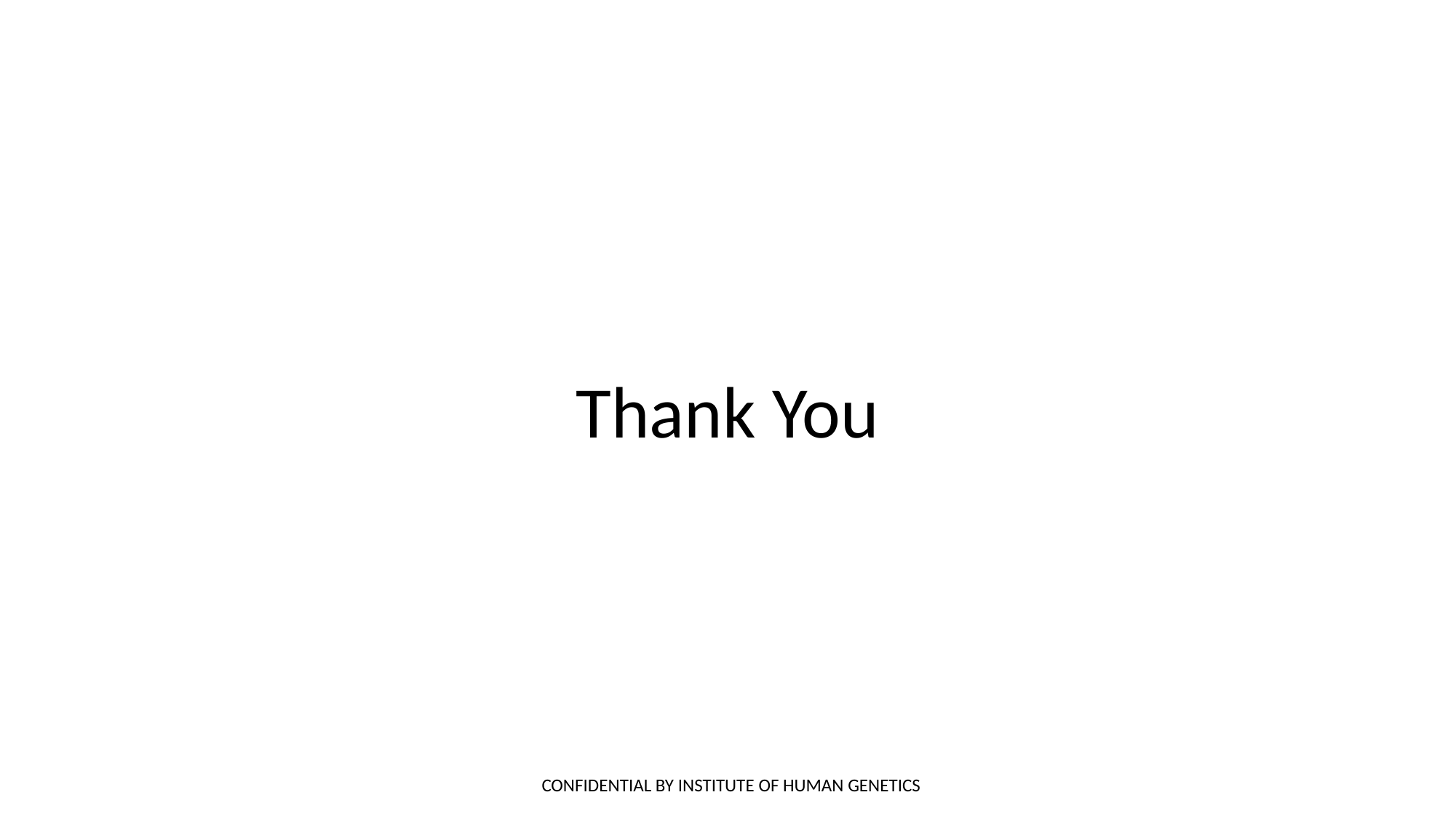

Thank You
